# NeuroDecon: A Neural Network-Based Method for Three-Dimensional Deconvolution of Fluorescent Microscopic Images

**DOI:** 10.3390/ijms26188770

**Published:** 2025-09-09

**Authors:** Alexander Sachuk, Ekaterina Volkova, Anastasiya Rakovskaya, Vyacheslav Chukanov, Ekaterina Pchitskaya

**Affiliations:** 1 Laboratory of Biomedical Imaging and Data Analysis, Institute of Biomedical Systems and Biotechnology, Peter the Great St. Petersburg Polytechnic University, Khlopina St. 11, St. Petersburg 194021, Russia; as.sachuk.bsns@gmail.com (A.S.); volkovakatusha04@gmail.com (E.V.); jonatepl@gmail.com (A.R.); chukanov_vs@spbstu.ru (V.C.); 2 Department of Applied Mathematics, Peter the Great St. Petersburg Polytechnic University, Polytechnicheskaya St. 29, St. Petersburg 195251, Russia; 3 Laboratory of Molecular Neurodegeneration, Institute of Biomedical Systems and Biotechnology, Peter the Great St. Petersburg Polytechnic University, Khlopina St. 11, St. Petersburg 194021, Russia

**Keywords:** fluorescence microscopy, deconvolution, deep learning, data generation, learning strategy, practical applications, data analysis

## Abstract

Fluorescence microscopy performance can be significantly enhanced with image post-processing algorithms, particularly deconvolution techniques. These methods aim to revert optical aberrations by deconvolving the image with the point spread function (PSF) of the microscope. However, analytical deconvolution algorithms are computationally demanding, time-consuming, and require precise PSF estimation and careful parameter selection for optimal results. This paper introduces NeuroDecon, a neural network-based method for volumetric deconvolution of confocal images with residual blocks and U-net based architecture. NeuroDecon employs a training strategy that implicitly incorporates the experimental PSF, which acts as a “fingerprint” of system aberrations. This open-source approach allows for personalized training dataset generation, enabling its wide usage for various applications, reduces imaging artifacts and improves computational efficiency. NeuroDecon network outperforms analytical deconvolution methods in image restoration, resolution, and signal-to-noise ratio enhancement and facilitates further data analysis with methods based on automatic segmentation, including protein cluster detection, endoplasmic reticulum network, and dendritic spine 3D-morphology analysis.

## 1. Introduction

Fluorescence microscopy is a specialized form of light microscopy that employs fluorescence to examine the structure and characteristics of both organic and inorganic specimens. This technique is extensively utilized in materials science, as well as medical and biological research. However, this method has certain limitations related to the wave properties of light and instrumental inaccuracies [1]: the image of an object obtained by microscopy is subject to aberrations and noise artifacts.

Since 1983, a multitude of mathematically formulated deconvolution algorithms aimed to neutralize aberrations introduced by the registration system have been developed [2,3]. Each of these methods has its own strengths and weaknesses. For example, some algorithms like the Wiener algorithm are fast but less accurate, and others, such as the Richardson–Lucy method, are more accurate but take longer to compute. These methods, with the exception of blind deconvolution techniques, require information about the defects of registration systems, usually in the form of the point spread function, the precise determination of which is crucial and determines the quality and accuracy of the result. The PSF can be calculated theoretically [4] or estimated experimentally, where the latter approach is more favorable despite being more challenging. It should also be noted that analytical deconvolution methods often require hyperparameters, such as the number of computation steps, for which the optimal values are unknown and must be determined through an exhaustive search through a specified subset of the hyperparameter space [5]. However, this process does not guarantee the quality of obtained results or optimal algorithm performance [1]. A logical step from analytical algorithm employment to fully neuronal network microscopy image processing era has been made when a combination of the traditional Richardson–Lucy iteration algorithm with a fully convolutional network structure (RLN [6]) was presented.

Neural networks have revolutionized the processing of images and videos in everyday life. These advancements can be translated into the scientific realm, particularly in the processing of microscopic images. Applying neuronal networks to the image restoration problem allows for quick processing of large datasets and alleviates the need for hyperparameter estimation. In addition, deep learning models can learn complex patterns and features from large datasets, leading to more accurate and detailed reconstructions of microscopic images. The drawbacks of such approaches that limit their use in practice are their dependence on large-scale datasets with coupled ground truth high-resolution and aberrated images, and the limited interoperability of pretrained models for other microscopy types and imaging conditions.

Considering everything mentioned above, we have developed a neural network for volumetric AI-deconvolution named NeuroDecon, which is based on U-net and residual blocks. Our method enhances resolution, especially along the *z*-axis, signal-to-noise ratio, and the level of details in images, and exceeds the performance quality and speed of analytical deconvolution. We also introduce a new training strategy for this network that uses the PSF implicitly without need for its determination by capturing the aberrations of each experiment from additional images, which may be easily acquired together with experiential data. We demonstrate the performance of NeuroDecon on synthetic datasets and diverse in vitro and in vivo biological samples acquired with live imaging, confocal, and expansion microscopy. Our method greatly enhances the accuracy of image quantitative analysis in many applications, including protein clusters detection, endoplasmic reticulum network, and dendritic spine 3D-morphology analysis.

## 2. Results

### 2.1. NeuroDecon Neuronal Network Architecture, Dataset Generation and Training Strategy

**Figure 1:**
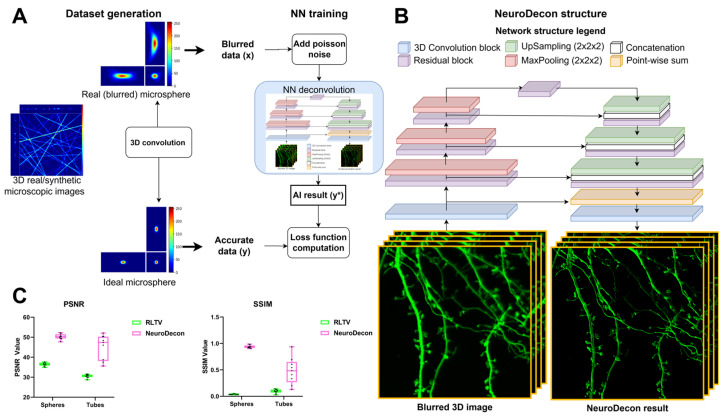
A neuronal network-based deconvolution of 3D fluorescent images with NeuroDecon: (**A**) NeuroDecon dataset generation and training procedure; (**B**) NeuroDecon architecture structure; (**C**) average PSNR and SSIM values on synthetic tubes and spheres dataset, *n* = 9.

To enhance the quality of three-dimensional (3D) z-stack images acquired from a confocal microscope, we have developed a fully convolutional 3D network named NeuroDecon. This network is based on two commonly used architectural practices for designing image-processing neural network structures: residual blocks and U-Net (Figure 1B). This design choice was motivated by the computational demands of processing 3D images. Step-by-step compression and decompression of feature dimensions enable efficient memory utilization [7]. In addition, the integration of residual blocks instead of the conventional multiple convolution layers at each layer of the U-Net image structure allows faster learning, mitigating the vanishing gradient problem [8]. Although developed for image recognition tasks, residual blocks have become a de facto standard for achieving more robust loss function convergence for a wide area of tasks. For a more detailed structure of residual blocks, see Section 4.2.1.

In addition to designing the neural network architecture, it is essential to develop a training procedure that ensures stable convergence of the loss function and create a training dataset. For training neural networks in image restoration tasks, it is crucial to acquire pairs of blurred images and their corresponding high-resolution counterparts, which serve as the ground truth. However, gathering such datasets is a time-consuming and resource-intensive process that significantly influences the performance of the neural network. We have created a novel training strategy that facilitates robust learning. The cornerstone of this strategy is an innovative data generation algorithm that enables the on-demand creation of extensive datasets, capturing all patterns associated with the blurring of observed objects in the particular registration system. This algorithm utilizes a convolution operation between an array of high-quality images of both blurred and accurately imaged fluorescent microspheres. This convolution process generates sets of blurred images, which are used for supervised learning, and accurate images, which serve as the expected output (Figure 1A). Additionally, Poisson noise with a randomly selected mathematical expectation from a predefined set of values is introduced into the blurred images. This step enhances the network’s robustness to potential noise in subsequent input data. The point spread function represents the response of the imaging system to a point source object and characterizes how it is blurred by the optical components. Fluorescent microsphere image which is close to a point light source object is approximating the PSF of the microscope. The process of fluorescent microsphere imaging and subsequent experimental PSF determination for the Richardson–Lucy Total Variation (RLTV) deconvolution algorithm used for comparison is described in Section 4.1.4. The accurate microsphere image (in ideal imaging conditions) was modeled using the ellipsoid equation and known imaging scale and sphere size values (refer to Section 4.1.3). The comprehensive process of data generation and training is illustrated in Figure 1A. This approach allows for accounting microscope inaccuracies by implicitly accounting for the PSF. The direct and often inaccurate calculation of PSF, which frequently results from poorly selected parameters, can introduce significant losses in subsequent deconvolution processes [1], and this approach overcomes that issue. This strategy also allows for precise control over the content of the training dataset by high-fidelity input data selection. This is crucial because deep learning-based deconvolution methods exhibit a strong dependency on the training data. In this case, the contents of the dataset can be controlled by adding a variety of structures and types of samples on which the neural network should operate during its use. For training dataset generation, high-resolution images obtained by an experimenter and collected from open repositories, or synthetically generated, can be used. The synthetic data can be obtained using algorithms that create various structures, such as spheres, tubes, surfaces, and tree-like or sponge-like structures, corresponding to the types of images planned to be processed with AI deconvolution. Moreover, we introduce the additional augmentation and processing of the original data which also improve the convergence of the model and allow for more robust learning, described in detail in [Supplementary Figure S1—The dataset generation pipeline]. The analytical justification of this data generation method and images datasets are described in [Section 4.2.3]. The training parameters of the network under various research aspects are presented in the table [Supplementary Tables S1 and S2—Datasets characteristics and Training parameters].

Deconvolution was performed using both the neural network method and the Richardson–Lucy with Total Variation Regularization method on a dataset comprising synthetic spheres and tubes (Figure 1C, Supplementary Figure S2) to evaluate the peak signal-to-noise ratio (PSNR) and structural similarity index (SSIM) metrics [9]. In addition, intensity plots along specific lines were generated to assess the accuracy of the recovered image structures. Synthetic data was used exclusively in the current experiment and were excluded from the training sample in subsequent experiments. A general overview of the images used is presented in Supplementary Figure S4.

The significant SSIM deviations observed in the RLTV method are primarily attributable to residual artifacts after noise addition, to which the SSIM metric is particularly sensitive, rather than to structural discrepancies. In contrast, the proposed NeuroDecon method does not show this drawback associated with the presence of noise. Moreover, as shown in [Supplementary Figure S3—NeuroDecon testing on synthetic noisy data], NeuroDecon is more robust than RLTV, as it is less sensitive to intensive input noise: this is especially evident in the increasing blurring along the OZ axis at high noise levels.

The PSNR deviations of the RLTV method, when compared to the NeuroDecon, are less pronounced than the deviations observed in the SSIM metric. This is explained by the presence of various structural inaccuracies, as illustrated in Supplementary Figure S2A with fragments of the original images and in Supplementary Figure S2B with intensity plots along specific lines. The NeuroDecon method does a better job of removing inaccuracies along the OZ axis. Furthermore, the details located at the intersection of the tubes are more apparent in the NeuroDecon result than in the RLTV result: this is evidenced by the more numerous peaks in the intensity plot, which correspond to the positions of the tubes.

The enhanced structural recovery achieved by NeuroDecon is presented in the fragment obtained from a real image of three fused fluorescent microspheres, as shown in Supplementary Figure S2C,D. The RLTV method fails to discern this feature, whereas NeuroDecon, leveraging its training sample, successfully restores the shape of that complex object. More results on real data with similar structures are illustrated in [Supplementary Figure S4A–C—NeuroDecon testing different tubes and confocal images]. All statistical analysis details are provided in Supplementary Table S3.

### 2.2. NeuroDecon Resolution Enhancement of Confocal Images Is Comparable with STED Super Resolution Microscopy

**Figure 2:**
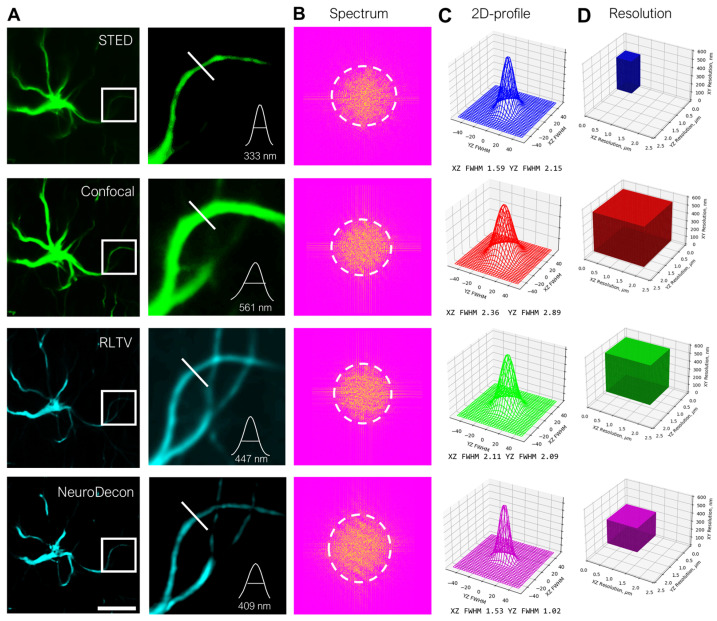
Demonstration of NeuroDecon for enhanced resolution and noise reduction in 3D-imaging: (**A**) from left to right, the full confocal images of astrocytes, enlarged views of the corresponding white boxes in the images with the intensity profiles along the corresponding white line; (**B**) the Fourier spectrum of the corresponding enlarged views in (**A**); (**C**) statistical 2D-profile plots of the astrocyte projections; (**D**) 3D resolution bar plots. The rows correspond to STED (first row), confocal (second row), RLTV (third row), and NeuroDecon (fourth row) methods of fluorescently labeled astrocyte. Scale bar: 10 µm for full images; 2 µm for enlarged views.

Super-resolution microscopy techniques offer high-fidelity biomedical imaging but are often inaccessible due to the need for expensive equipment, advanced technical expertise, and complex specimen preparations. We present NeuroDecon as a possible alternative to such techniques that can greatly improve and facilitate biomedical research. To demonstrate its ability to compare with super-resolution techniques, we assessed the performance of NeuroDecon on biological data. First, we used fixed astrocytes stained with anti-GFAP mouse monoclonal antibodies for comparative visualization with STED super-resolution and confocal microscopy. Representative images (Figure 2A, first column) of fluorescently labeled astrocytes were analyzed, with enlarged views highlighting resolution differences (Figure 2A, second column). Fourier spectrum analysis and average astrocyte projection profile plots (Figure 2B,C) quantitatively demonstrated substantial resolution enhancement by the STED and NeuroDecon methods, and measured Kc (cutoff frequency) and 2D-profiles indicate their superior ability to resolve fine structural details compared to confocal imaging and RLTV-deconvolution. Statistical analysis revealed that NeuroDecon provided 2× lateral and 1.5× axial resolution improvements (Figure 2D, Supplementary Figure S5) with minimal artifacts, with the greatest difference in the XY plane. RLTV deconvolution has shown an improvement in resolution across XZ and YZ planes, with little effect on the XY plane. This method demonstrated advantages of NeuroDecon over the RLTV approach, offering an efficient and versatile alternative for image processing. It was also shown that RLTV deconvolution, while capable of enhancing resolution, lowers the signal-to-noise ratio of the image significantly, while NeuroDecon improves it (Supplementary Figure S5), allowing for clearer interpretation of the biological data. The performance of NeuroDecon was also assessed on fluorescent microspheres as an example of the performance of the method on a standardized research object (see Supplementary Figure S8). Statistical analysis details for all metrics are provided in Supplementary Table S3. These results demonstrated that NeuroDecon deconvolution exhibited a balanced performance, providing competitive to hardware-based methods of resolution enhancement and superior noise reduction.

### 2.3. NeuroDecon Improves Resolution and Reduces Noise in Expansion Microscopy

**Figure 3:**
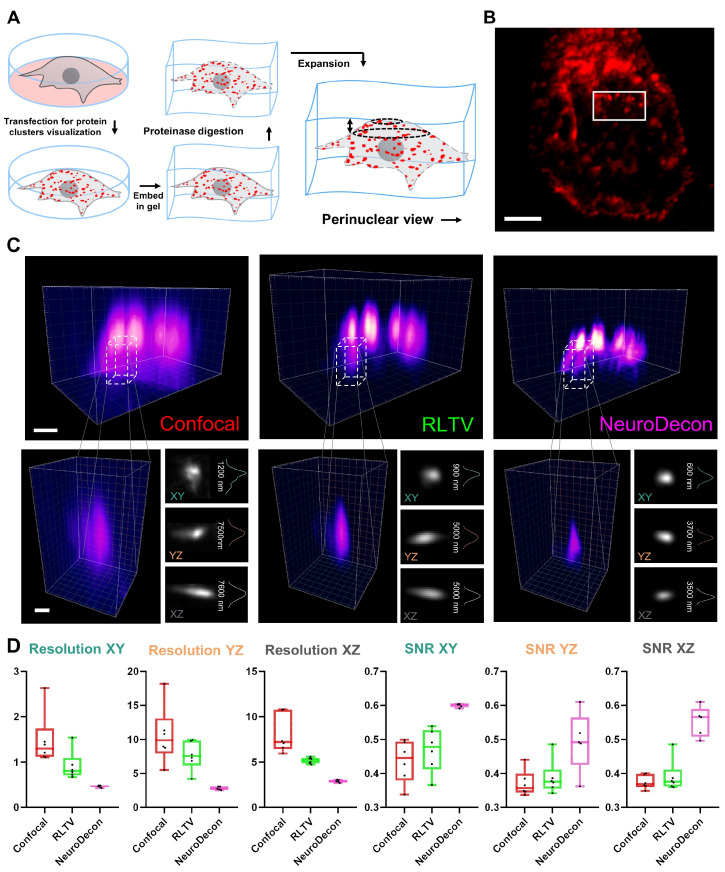
Improving resolution and reducing noise in ExM with NeuroDecon: (**A**) a schematic depiction of ExM sample preparation procedure; (**B**) a raw confocal image of STIM clusters in the HEK239T cell. Scale bar corresponds to 5 µm; (**C**) from left to right, 3D-reconstructed clusters from the boxed region in (**B**) of an unprocessed image, the results of RLTV and NeuroDecon. The bottom row depicts singular clusters and their projections with corresponding FWHM plots. Scale bar corresponds to 2 µm for top-row images and 1 µm for bottom-row images; (**D**) statistical comparisons of confocal images, RLTV deconvolution, and NeuroDecon for signal-to-noise ratio (SNR) and resolution metrics across the XZ, YZ, and XY planes, *n* = 6. Source data for all experiments are provided with this manuscript and summary statistics are provided in Supplementary Table S3.

Expansion microscopy (ExM) [10,11] is an inexpensive and powerful tool for studying super-resolved tissue microstructures using conventional confocal microscopes. The method provides locally isotropic expansion with minimal distortion (Figure 3A), improving both lateral and axial resolution by several factors (approximately 4×) [10] with iterative techniques reporting up to 20× [12]. However, when imaging extended samples, certain challenges persist, including the need to enhance the signal-to-noise ratio (SNR) and reduce scattering. NeuroDecon has been shown to significantly improve the quality of the images acquired with the use of ExM. We performed NeuroDecon deconvolution on expanded HEK293T cells, transfected to overexpress STIM1 protein forming clusters and stained them with the primary anti-mCherry antibody to enhance the signal (Figure 3B). Representative images revealed that the NeuroDecon approach provided the clearest and sharpest details, with distinct features visible across all cross-sectional slices. In comparison, RLTV demonstrated moderate improvements over confocal microscopy. Confocal imaging, while foundational, struggled to achieve comparable resolution and exhibited significant noise interference (Figure 3C). Quantitative analysis confirmed that the NeuroDecon method consistently achieved the highest resolution across all planes, with minimal variability, outperforming RLTV and confocal microscopy (Figure 3D). Similarly, the NeuroDecon method demonstrated superior SNR, excelling in noise reduction while preserving signal fidelity. Similar analysis has been conducted on in vivo ExM images, which have a significantly higher background noise level, with NeuroDecon presenting favorable results (see Supplementary Figure S9). Statistical analysis details for all metrics are provided in Supplementary Table S3. Overall, this comparison underscores the advantages of combining expansion microscopy with NeuroDecon.

### 2.4. NeuroDecon Improves Live- and Fixed- Cell Confocal Image Quality to Reveal Intricate Organelle Structure

**Figure 4:**
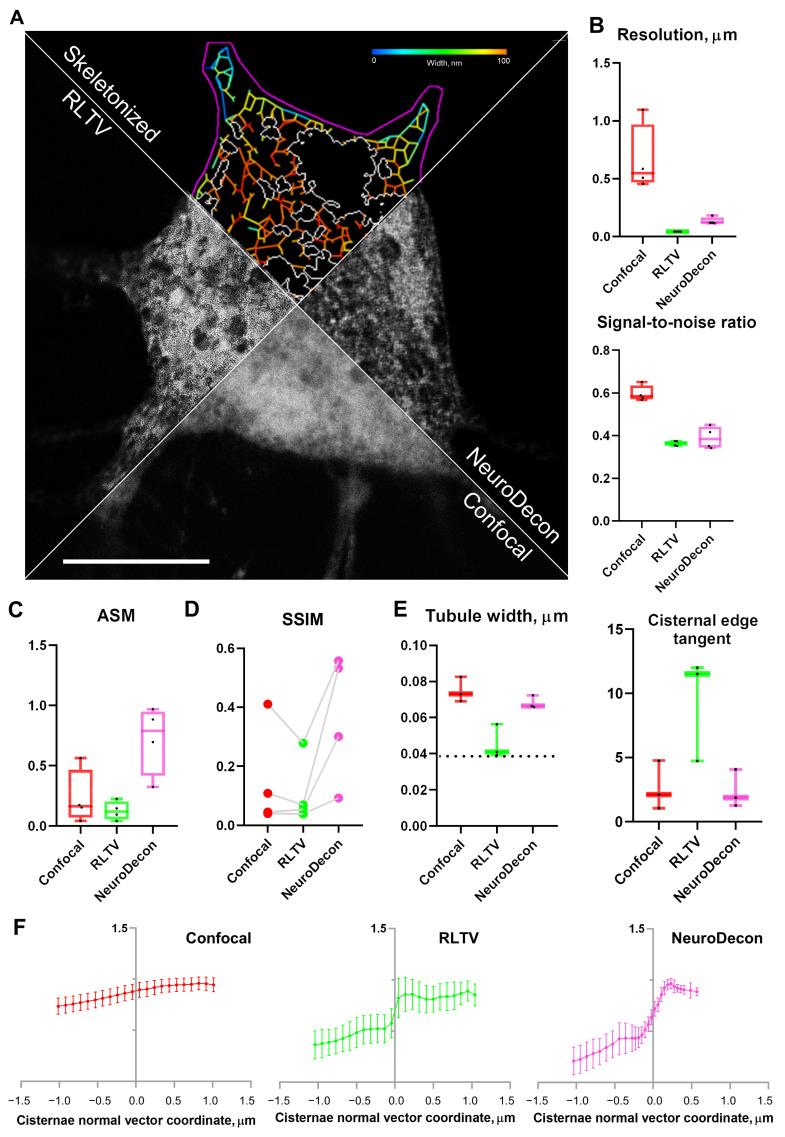
NeuroDecon improves live confocal image quality to reveal intricate organelle structure. (**A**) A representative image of unprocessed neuronal ER (bottom), skeletonized ER (top), RLTV results (left side), and NeuroDecon results (right); (**B**) top to bottom, statistical comparisons of confocal images, RLTV deconvolution, and NeuroDecon for XY resolution (*n* = 4) and signal-to-noise ratio (*n* = 4) metrics; (**C**) statistical comparison of confocal images, RLTV deconvolution, and NeuroDecon for Angular Second Moment (*n* = 4); (**D**) statistical comparison of structural similarity index metric for confocal images, RLTV deconvolution, and NeuroDecon (*n* = 4); (**E**) statistical comparisons of confocal images, RLTV deconvolution, and NeuroDecon for tubule width (*n* = 3) and cisternal edge tangent (*n* = 4), dash line corresponds to the minimal physiological tubule width; (**F**) average intensity profiles of cisternal edges of confocal images, RLTV deconvolution, and NeuroDecon. Source data for all experiments are provided with this manuscript and summary statistics are provided in Supplementary Table S3.

Live confocal imaging is a family of methods that aim to capture the physiological processes that occur in living cells and tissues and to reveal the morphology of organelles that are sensitive to fixation [13]. This method, while providing physiological results, has certain technical issues associated with it, such as weak fluorescence of the samples. The obtained images are dull and have a low signal-to-noise ratio, with fine intracellular structures, such as the endoplasmic reticulum (ER) structural components, heavily obscured by noise. While some methods, such as texture analysis, can be sensitive enough to detect changes in ER morphology [14], the more widespread tactics require careful image pre-processing. Applying NeuroDecon and RLTV methods to the images of live neuronal ER visualized with DsRed-ER transfection has revealed that the results of both deconvolution methods allow for visual differentiation of structural parts of ER. To further investigate the quality of image restoration, automatic segmentation of ER parts was performed with AnalyzER [15]. The representative images and the corresponding segmentation are depicted in Figure 4A. While RLTV deconvolution has produced images with higher resolution than NeuroDecon (Figure 4B), the associated “graininess’’ of the image, which is quantitatively shown as a decrease in the Angular Second Moment (ASM) of the picture (Figure 4C), has become detrimental to proper ER analysis. In particular, the loss of image homogeneity hindered proper tubule segmentation (Figure 4E) and obscured the contours of ER cisternae (Figure 4F). The images processed with NeuroDecon have shown to have higher ASM and allowed for acquisition of biologically sensible data (Figure 4E,F). To ensure that image restoration with NeuroDecon did not misrepresent the structure of the tubule network, raw confocal images were skeletonized, and the structural similarity index (SSIM) for deconvolution results was measured, with NeuroDecon showing better results than analytical deconvolution (Figure 4D). To ensure that inherent variations in tubule width did not significantly affect calculation results, additional region-specific measurements were taken (See Supplementary Figure S10). Additional analysis has confirmed that the results of NeuroDecon deconvolution have a higher root-mean-square-error between them and the neuronal growth cone confocal images than the results of RLTV deconvolution, while simultaneously remaining strongly correlated to them (See Supplementary Figure S11), suggesting little to no artifacts [16,17]. Another experiment aimed at assessing the compatibility of NeuroDecon with machine learning-based image analysis was conducted. Dendritic spine images (in vitro, in vivo and after expansion microcopy) were processed with NeuroDecon and analyzed with SpineTool (version 4.3.29) [18] (See Supplementary Figure S12). In this experiment, NeuroDecon has also been shown to improve image quality for all input data, thus facilitating further quantitative analysis. Statistical analysis details for all metrics are provided in Supplementary Table S3. Overall, this comparison highlights the advantages of using NeuroDecon for intricate cell and organelle morphology investigation.

### 2.5. NeuroDecon and Similar Existing Deep Learning Method for Image Restoration

**Figure 5:**
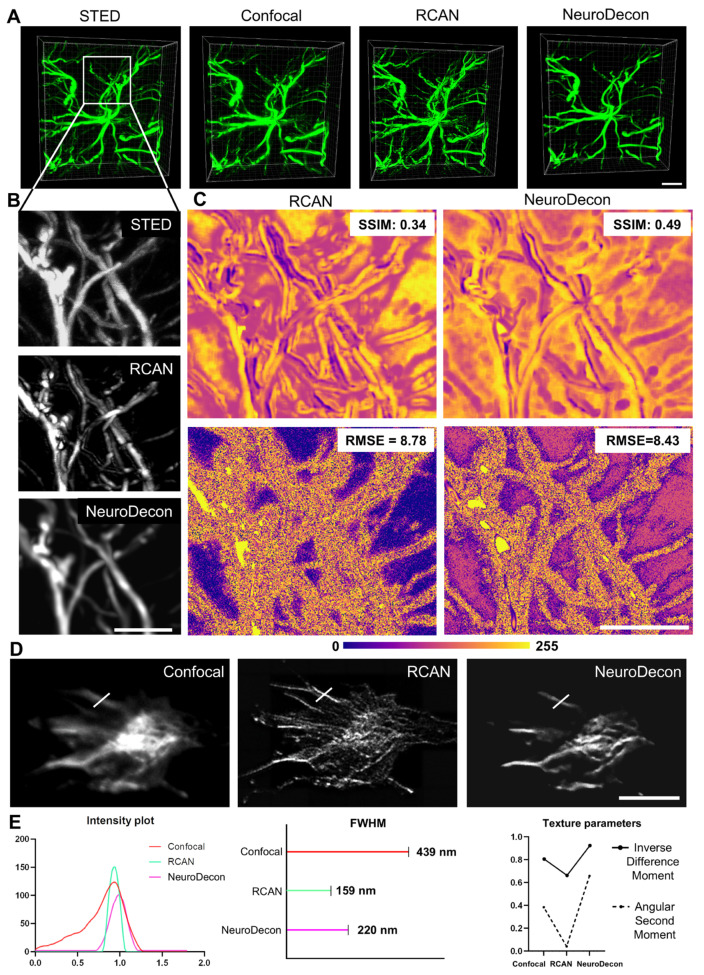
NeuroDecon and similar existing deep learning methods for image restoration: (**A**) representative 3D-restorations of astrocytes. From left to right, a restoration of STED image, unprocessed confocal image, RCAN, NeuroDecon deconvolution. Scale bar corresponds to 7 µm; (**B**) top to bottom, enlarged views of astrocyte projections of STED image, RCAN, NeuroDecon deconvolution. Scale bar corresponds to 5 µm; (**C**) top row: local SSIM maps and global SSIM metric for NeuroDecon-to-STED and RCAN-to-STED comparisons. Bottom row: NeuroDecon-to-STED and RCAN-to-STED error maps with global RMSE metric. Scale bar corresponds to 5 µm; (**D**) representative images of growth cones. From left to right, an unprocessed confocal image, RCAN, NeuroDecon deconvolution; (**E**) from left to right, intensity plots from the area depicted as a white line in (**E**), FWHM of these intensity plots, texture metrics for representative images in (**D**). Scale bar corresponds to 5 µm. Source data for all experiments are provided with this manuscript and summary statistics are provided in Supplementary Table S3.

As neural network-based deconvolution methods are a promising alternative to analytical methods, several networks for image quality restoration have been developed over the last few years. To highlight the advantages of our method, we present a comparative analysis of NeuroDecon and 3D-RCAN [19] performance, as 3D-RCAN is a deep learning method most closely related to our work. Unlike other methods that solve super-resolution or 2D deconvolution problems [6,19,20,21,22], 3D-RCAN operates without scale change in the output. Notably, 3D-RCAN exhibits several principal differences from our proposed solution. Firstly, the network architecture of 3D-RCAN does not change features sizes with upsampling and downsampling layers, whereas our method employs a modified U-Net architecture. Secondly, NeuroDecon uses a modified error function that not only focuses on the error between the outputs expected and received by the network but also takes into account the content of the network output. Lastly, the generation of training data in 3D-RCAN relies on an explicit definition of the PSF, which may introduce inaccuracies in the future generation; conversely, our method allows training without explicitly defining the PSF, but captures unique imaging system properties and unsymmetricity. Figure 5 illustrates a comparative performance evaluation of 3D-RCAN and NeuroDecon on two image types: astrocytes captured via confocal microscopy and STED (Figure 5A–C, Supplementary Figure S13), and growth cones captured via confocal microscopy (Figure 5D,E). As is evident in the representative images, NeuroDecon enhanced image resolution more effectively than 3D-RCAN, not misrepresenting the structure of astrocyte projections. It is further supported by Figure 5C where a comparison of the deconvolution results with STED results is shown as SSIM and error maps (top and bottom rows, respectively). These maps illustrate the superior signal restoration and noise reduction capacity of NeuroDecon, as the structural similarity is higher in the regions of interest and the error is higher in background areas. Another experiment shows that NeuroDecon performed similarly to 3D-RCAN in terms of SNR and resolution metrics (Supplementary Figure S13).

Growth cone image analysis showed qualitative (Figure 5D) and quantitative (Figure 5E) differences in the methods performance. NeuroDecon increases the homogeneity of tubular structures, while 3D-RCAN yields more “jagged”, “grainy” results. Quantitatively it is estimated as lower contrast (as higher values of homogeneity, or Inverse Difference Moment) (Figure 5E) of NeuroDecon results compared to RCAN results. The 3D-RCAN results have also shown to be more disordered, as is illustrated by lower Angular Second Moment in Figure 5E. NeuroDecon, however, lacks sensitivity and cannot restore the dimmest parts of the image, while 3D-RCAN excels in highlighting finer details, thinning the lines and making the edges sharper.

The aforementioned limitations stem from inherent shortcomings in both algorithms: in the case of NeuroDecon, an unsuccessful selection of the error regularization hyperparameter. This issue is pervasive in many deconvolution methods, manifesting in aspects such as PSF definition accuracy, network parameter selection, and error function design. To ensure an unbiased evaluation, we present in this comparison the performance results after only one single iteration of training with a single choice of hyperparameters for both methods. As can be seen from our previously discussed results (Supplementary Figure S2, Figure 4D), optimal hyperparameter selection enhances structural preservation accuracy. All statistical analysis details are provided in Supplementary Table S3.

## 3. Discussion

We propose a deep learning method designed to enhance the quality of 3D images acquired through fluorescence microscopy by eliminating blurring artifacts. Additionally, our proposed data generation algorithm facilitates the adaptation of this method to the actual PSF of various microscopes with diverse imaging settings. Notably, the algorithm generates data without the need for explicit definition of the PSF, which is particularly beneficial in scenarios where modeling the PSF is challenging. Moreover, this algorithm does not require additional ground truth image acquisitions for each blurry image, which are generated at additional cost and effort. The inclusion of Poisson noise in the input data of the training dataset enables the method to deconvolve images containing negligible noise without the need for pre-denoising (Figure 1, Supplementary Figure S2). The efficacy of this method has been demonstrated through a series of measurement experiments on restoration of various biological data, where NeuroDecon has shown to produce accurate results with minimal artifacts that may be directly used to draw biological conclusions or subjected to further processing with other software (Figure 2, Figure 3, Figure 4 and Figure 5). We anticipate that this research will be widely adopted in most labs to replace analytical deconvolution, enabling fast and effective improvements in routine visualization without the need for costly super-resolution microscopy or other hardware-based resolution enhancement techniques.

Several variants of the problem of improving microscopy image quality exist. One notable example is the denoising problem. Although similar in formulation and objectives to deconvolution, it differs significantly in its formal structure. Instead of addressing blur, which can be mathematically represented by convolution, denoising focuses on eliminating various types of noise, such as Poisson noise. In NeuroDecon, the addition of the noise to the training dataset partially solved the denoising problem, reducing it in the output and making that approach more robust to potential noise in the samples.

Another related task is enhancing resolution by increasing the number of points in a raster image of an object. This problem also aims to improve image quality, but it does so by augmenting the number of data points, which can be viewed as a generative process. While this problem is more closely aligned with our objectives compared to denoising, it presents unique challenges and underlying assumptions. Consequently, it was not considered in our study.

There are various neural network methods that enhance microscope images through denoising or resolution enhancement available up to date [6,19,20,21,22,23,24,25]. The predominant research trend in addressing this problem involves exploring methods to leverage networks from computer vision and modifications of their training procedures. For instance, the CARE [22] method employs a U-Net architecture to provide image denoising and size increasing using upscaling, demonstrating the enhancement of image size and quality for both two-dimensional (2D) and three-dimensional (3D) images. U-Net has also been applied in other methods; for example, it is used for resolution enhancement in ZS-DeconvNet [23] and DFCAN [21]. These studies focus on modifying training procedures using Generative Adversarial Networks (GANs) and validating their effectiveness across different microscopy types (SIM, Wide-Field, Confocal, and TIRF). Another example is the new DeAbe [25], which, like the ZsDeconv [23] method, also focuses on enhancing image resolution with different types of aberrations. Furthermore, other network architectures have been investigated. The Richardson–Lucy network (RLN [6]) has been presented, which combines the traditional Richardson–Lucy iteration with a fully convolutional network structure. Notably, channel attention mechanisms, implemented through the RCAN [19] network, have been successfully applied not only to address image size enhancement but also to solve the conventional 3D deconvolution problem.

The objectives of the RCAN method are most closely aligned with ours; however, in the RCAN paper the results presented have been acquired using explicitly constructed PSF. In contrast, our study investigates the application of neural network methods to microscopes with aberrations, without the need for labor-intensive procedures to reconstruct PSF (an example of the consequences of such aberrations can be observed in the image of a sphere depicted in Figure 1A). As mentioned in DeAbe [25], where a mixture of random low-order aberrations is used for model training, performance enhancement is anticipated if aberrations specific to the sample are used in model training. Our approach, which involves capturing the image of a small round fluorescent bead that closely resembles the real system’s PSF, can capture the unique aberrations of the current experiment introduced by both the specimen and the hardware setup. The future direction could involve developing AI-based methods for estimating the experimental PSF directly from the experimental image volume once a sufficient dataset of PSF-image pairs and a theoretical foundation for such work have been established.

The current design of NeuroDecon network allows for enhanced data collection from the obtained experimental images and its relevant and accurate biological interpretation. It enhances the signal-to-noise ratio of the images and improves resolution, as supported by decorrelation analysis. Minimal artifacts related to the spatial intensity distribution (i.e., minimal darker or lighter spots within the areas of the original image with even intensity) allow for seamless import of experimental data into machine learning-based analysis algorithms. An example of such a pipeline would be automated segmentation of dendritic spines or structurally different parts of endoplasmic reticulum after NeuroDecon employment. These kinds of biological problems tend to be nearly unresolvable with poorer-quality input data, yet NeuroDecon application could potentially significantly lower the quality demands, facilitate research, and provide more data that has been otherwise obscured.

Notwithstanding the achieved image quality enhancements, this method exhibits several limitations due to its solution construction and the general nature of deep learning techniques. Firstly, the method’s performance is contingent upon the data present in the training sample. Despite the optimized data generation process, the method may not accurately enhance images of objects with structures differing from those in the training dataset. Secondly, to obtain the most accurate results, the trained model must be applied to images with blurring characteristics matching those in the training sample. Nevertheless, the proposed strategy for generating training datasets is easily adaptable to new types of images without the need for acquiring ground truth data. This observation, combined with the rapid convergence of the model, enables research groups to efficiently develop a set of frequently used models, named ”model zoo”, tailored to their specific research tasks and experiments. These models not only align closely with their research objectives but also facilitate the acquisition of high-quality images under challenging PSF conditions. Consequently, repeated training is required for each new set of imaging device parameters that define a new PSF. Lastly, the method’s performance is dependent on the selection of a hyperparameter—the regularization parameter of the loss function, whose optimal value is challenging to determine, despite the method’s rapid convergence (see Supplementary Figure S6).

These limitations, which may impede researchers’ work due to the additional costs associated with retraining, are prevalent among many of the methods discussed in this paper. These challenges delineate a new frontier of research questions that remain to be addressed, namely the following: how to develop a method that is robust to variations in the PSF, how to accelerate the convergence process, and how to make this process independent of the data present in the training dataset. After accumulating user experience and developing advanced image analysis approaches, the opportunity to create a foundational model for enhancing microscopic images across a wide range of applications will be opened, combining deconvolution, denoising, and upscaling for various types of the microscopy.

## 4. Materials and Methods

### 4.1. Image Synthesis

#### 4.1.1. Spheres Synthesis

In order to perform a qualitative evaluation of the algorithm’s performance, as well as to generate data for training when there is a limited amount of real accurate data, it is useful to be able to generate synthetic data. The developed algorithms of different kinds of structure generation are provided.

For volumetric spheres images generation, the ellipsoid equation was used as follows:

(x − x_c_)^2^/r_x_^2^ + (y − y_c_)^2^/r_y_^2^ + (z − z_c_)^2^/r_z_^2^ <= 1

where (x_c_, y_c_, z_c_) are the coordinates of the center of the ellipsoid in the image: i_c_ ∈ Ν∪{0}, i ∈ {x, y, z}; (r_x_, r_y_, r_z_) are the scales along different three axes: r_i_ ∈ R\{0}, where i ∈ {x, y, z}.

This formula determines only if a point of the image is inside of the ellipsoid: if for some point of the image (x, y, z) the inequality is satisfied, then this point belongs to the ellipsoid, otherwise it does not. Varying the intensities of points inside the ellipsoid by a uniform discrete distribution, the scales r_i_ by a uniformly continuous law, and the coordinates of the center inside the image, it is possible to obtain synthetic images containing spheres of different shapes, sizes, and brightnesses.

#### 4.1.2. Tubes Synthesis

To generate more various synthetic data, rather than only spheres, we provided generation of synthesis tubes. The tube generation algorithm depended on variables like intensity, size, point of volumetric image, which must be in the tube, and some angles of the directing vector of the tube. By choosing these parameters by uniform continuous distribution, we provide variative and uniform generation of possible lines, which can be observed on synthetic images. This property is important in dataset generation. More info about this algorithm was written in Supplementary—see Synthetic tubes generation.

The result of tubes and spheres synthesis is presented in ‘Neuro-Decon-data.zip’ Supplementary Materials (see ‘synthetic_data’ folder).

#### 4.1.3. Accurate Bead Calculation

For accurate bead calculation, an algorithm synthetic sphere was used. Providing the sphere radius as half of the diameter and scales along different axes, we can find points of the image that will belong to the sphere.

#### 4.1.4. Blurred Bead Calculation

For dataset generation and, as a consequence, for model training, blurred bead must be calculated. For solving this task, images of spheres are captured with methods which were described above. After that, from the captured volumetric image, single placed spheres (or beads) are manually extracted by the researcher. Then, the set of single beads are averaged by the centers of the beads: using the big number of spheres leads to removing some noise.

For finding bead center, each image of single bead was blurred with Gaussian blur with sigma σ = 3 and the brightest point of image was taken as a center. It is necessary to mark that blurred images of beads were used only for center finding: in image averaging original images of single spheres were used.

The blurred spheres were employed in the experimental determination of point spread functions (PSFs) for subsequent deconvolution using the Richardson–Lucy Total Variation (RLTV) method. Various hyperparameter configurations were evaluated; however, the optimal configuration for our experimental setup and registration systems comprised 10 iterations of the method with a regularization parameter of λ = 10^−5^. The software utilized for PSF extraction was the “DeconvolutionLab2” plugin for ImageJ (version 2.1.2), consistent with other deconvolution procedures. Further details are provided in Section 4.3. A sphere was employed as a precise reference, with its modeling detailed in the section titled “Section 4.1.3”.

### 4.2. Model Architecture and Training

#### 4.2.1. Model Description

The deep learning model NeuroDecon (code available in Supplementery, see ‘Neuro-Decon-code.zip’) is a modification of the original U-Net network: the modification consists of replacing pairs of layers with residual blocks. Each residual block has three layers of 3D convolution with LeakyReLU activation layers going after each of them. The slope parameter of the straight line on the negative part at the LeakyReLU layer was 0.3. The size of the 3D convolution filters in the residual blocks was chosen to be 3 × 3 × 3; in the other layers that are placed in the input and output of the network it was 1 × 1 × 1. The latter type of layer was designed to decrease or increase the number of channels of the feature map. The structure of the described residual block is shown in Supplementary Figure S7.

In the original U-Net network architecture, the number of channels increased by two after each downsampling with the MaxPooling layer and decreased by two after each transposed convolution layer. In NeuroDecon network, the transposed convolution layers have been replaced by UpSampling layers. The first residual block has 16 channels as input. Then, after each MaxPooling3D layer, the number of layers in subsequent residual blocks is doubled; after upSampling layers, the number of layers is halved. The number of downsamplings and upsamplings was chosen to be three.

#### 4.2.2. Dataset Generation Mathematical Justification

Consider a set of volumetric images {i_k_}^N^_k=1_, which can either be generated algorithmically or acquired from a microscope. To create training datasets, we utilize blurred spheres o and exact spheres i, generated according to Section 4.1.3 and Section 4.1.4. These spheres are fitted to convolution filters i* and o* such that the sum of the intensities of all points in both i and o equals to 1. We assume that the blurred sphere o* is the result of the convolution of the exact sphere i* with the PSF p of the capturing system:

o* = i* ⊛ p.

By convolving the images {i_k_}^N^_k=1_ with filters i* and o*, we obtain two sets of images, X and Y, which constitute the dataset for training:

X = {x*_k_* = i_k_ ⊛ o*} ^N^_k=1_,

Y = {y*_k_* = i_k_ ⊛ i*} ^N^_k=1_.

The dataset (X, Y) enables the training of a neural network to perform deconvolution without explicitly determining the PSF p. This is feasible due to the associative property of convolution: the images in X are essentially the convolutions of the images in Y with PSF p:

x*_k_* = i_k_ ⊛ o* = i_k_ ⊛ (i* ⊛ p) = (i_k_ ⊛ i*) ⊛ p = y_k_ ⊛ p.

This methodology generates the necessary datasets for training. The details of how the images o* = i* ⊛ p were obtained to create the dataset are described further in Section 4.2.3.

#### 4.2.3. Dataset Generation Procedure

To generate a set of volumetric fixed-size images {i_k_}^N^_k=1_ we utilized large images of various organic structures, including astrocytes, neuronal spines, endoplasmatic reticulum, and growth cones. These large images were segmented into fragments of a fixed size, which were then subjected to various augmentation techniques such as rotations, noise addition, and structure magnification. Consequently, a set of augmented fragments {i_k_}^N^_k=1_ was formed.

For a more detailed description of the entire dataset generation algorithm, please refer to Supplementary Figure S1. For more details about dataset characteristics, please refer to Supplementary Table S1.

#### 4.2.4. Blurred Bead Calculation

For model training, the developed loss function, which is based on an MSE with Hessian regularization parameter, was used for minimization:

L(I,I*) = ∑*_n_* (|I_n_ − I_n_*|)/N + α∑*_n_* (R_Frob_(Hessian(I))*_n_*)/N

where I, I* are images predicted by the network and the accurate images, N is the number of points on the image, *n*—variable for iteration, R_Frob_(∙)—Frobenius norm, Hessian—method for counting discrete second derivatives of predicted image, α—regularization term.

The mean of the Frobenius norm of second derivatives made the images look less torn and more complete, especially for some of the images with an already known structure.

The optimizer used was AdamW, which is an Adam optimizer [26] with L2 regularization of the network weights. The regularization parameter of the weights is equal to 0.004, β_1_ = 0.9, β_2_ = 0.999, ε = 1 × 10^−7^. Model training was performed with version 2.15 of Tensorflow library [27].

Datasets were divided into training and tested at a ratio of 4:1 (80% and 20%). The model was trained for 200 epochs on computational power 1 GPU A100. It should be noted, however, that the trained model can be deployed on V100. The time of one epoch learning on the training dataset was 6 min. Total learning time was above 24 h. However, the convergence of the error function to the constant was observed strongly in advance, at about 50–100 epochs [see Supplementary Figure S6—Average training results]. For more details about learning procedures for every experiment, please refer to [Supplementary Table S2—Training parameters].

### 4.3. Other Deconvolution Methods

#### 4.3.1. Richardson–Lucy Total Variation

To compare the developed neural network with traditional analytical methods, the Richardson–Lucy Total Variation method was used. This method was chosen because it is estimating the object by considering the nature of the noise, similar to our neural network. For this paper, “DeconvolutionLab2” plugin for ImageJ was used [28]. For each image, different values of regularization term and different numbers of iterations were provided (Figure 1, Figure 2, Figure 3, Figure 4 and Figure 5).

#### 4.3.2. 3D-RCAN

To compare the proposed method with 3D-RCAN, we utilized the code and instructions provided by the authors (https://github.com/AiviaCommunity/3D-RCAN, accessed on 24 August 2025). Initially, 3D-RCAN training included data augmentation through rotations. However, since our data are sensitive to rotations due to asymmetric PSF aberrations, we modified the original solution by disabling these augmentations. Other parameters, such as the number of residual groups and blocks, the number of epochs, and steps per epoch, remained unchanged. For training 3D-RCAN, datasets were generated following the procedure outlined by the authors. To create the input data, sets of large, accurate images were convolved with PSFs determined using the Richardson–Lucy method. The output data consisted of the same large, accurate images prior to convolution with the PSFs. The organic structures used to generate the datasets for both NeuroDecon and 3D-RCAN were identical (Figure 5).

### 4.4. Sample Preparation

Albino inbred mice (FVB/NJ, strain #001800), line M mice Tg (Thy1-EGFP) MJrs/J (strain #007788), 5xFAD mice (strain #034848) were obtained from the Jackson Laboratory (Bar Harbor, ME, USA). These mice were established and maintained in a vivarium, with four to five mice per cage and a 12 h light/dark cycle in the animal facility. Food and water were available ad libitum. All P0 mice pups used in experiments were euthanized by decapitation with sharp surgical scissors. All studies correspond to the principles of humane treatment of animals and were approved by the Bioethics Committee of Peter the Great St. Petersburg Polytechnic University in St. Petersburg, Russia, and also followed the principles of European convention (Strasbourg, 1986) and the Declaration of the International Medical Association for the Humane Treatment of Animals (Helsinki, 1996). All methods were carried out in accordance with relevant guidelines and regulations. The study was carried out in compliance with the ARRIVE guidelines (Figure 2, Figure 3, Figure 4 and Figure 5).

Rat brain tissue from animals weighing 150–180 g and 0.5 years old was studied. After anesthesia (xylazine and zoletil), the animals were perfused transcardially with phosphate buffer and 4% paraformaldehyde at 37 °C. The brain was then post-fixed overnight in paraformaldehyde at 4 °C. Areas studied included the barrel cortex (S1), thalamic nuclei (VPM, VPL, RT), and hippocampus. Frontal 40 µm sections were cut using a vibratome, identified under a stereotactic loupe, and then cryoprotected in sucrose solutions. Sections were incubated with primary antibodies to GFAP (Sigma-Aldrich, St. Louis, MO, USA, SAB5201104), followed by secondary antibodies goat anti-mouse conjugated with Abberior STAR RED (Abberior, Heidelberg, Germany). Visualization was performed using a high-resolution system (Abberior Facility Line, Abberior Instruments GmbH, Germany) in confocal laser scanning mode and STED mode (Figure 2 and Figure 5).

To analyze STIM1 clusters in HEK-293T line cells (Cell Culture Collection, Institute of Cytology, Russian Academy of Sciences, St. Petersburg, Russia) with 50–70% of confluency, cells were transfected with the Cherry-STIM1-TR/NN (was kindly provided by Dr. Jen Liou (University of Texas Southwestern Medical Center, USA)) plasmid using a polyethylenimine reagent (Polysciences Inc., Warrington, PA, USA, #23966) in serum-free Opti-MEM medium (Thermo Fisher Scientific, Waltham, MA, USA, #11058-021). Then, samples were expanded in accordance with the protocol of expansion microscopy [29] to enlarge the sample and visualized using confocal microscopy (0.072 µm/pixel, 2048 × 2048 pixels) (Figure 3).

Primary hippocampal cultures of dissociated hippocampal cells were prepared from newborn FVB mice on 0–1 postnatal day; three to five mice were used to obtain one culture as in details described in [30]. Briefly, after removing meninges and cerebellar tissues, hippocampi were digested in papain solution for 30 min at 37 °C (3176, Worthington, Columbus, OH, USA), triturated in 1 µg/mL DNAseI (DN-25, Sigma), and pipetted. After centrifugation, supernatants discarded and fresh, warm (37 °C) growth medium (Neurobasal-A (Gibco, Waltham, MA, USA, 10888), B-27 (17504, Gibco), 1% FBS, 0.5 mM L-Glutamine (25030, Gibco)) was added. Neurons were plated in 24-well plates containing 12 mm round Menzel cover slips (d0-1) precoated with 1% poly-D-lysine (p-7886, Sigma) (Figure 4 and Figure 5, Supplementary Figure S9).

To detect growth cones from neuronal culture at DIV2, the cells were fixed with 4% paraformaldehyde in PBS for 10–15 min at room temperature and staining was performed using rhodamine-phalloidin (#1475357, Thermo Fisher Scientific) for 10 min in the dark at room temperature. For assessment of the neurin growth cones, a Z-stack of the optical section was captured with a confocal microscope (Leica TCS SP8, Nussloch, Germany). For growth cones, 2048 × 2048-pixel images with a 0.0189 µm/pixel resolution were captured with Z interval of 0.2 µm using a 100× objective lens (NA = 1.4, UPlanSApo; Olympus, Tokyo, Japan) (Figure 4 and Figure 5).

To detect dendritic spines at DIV7, transfection was performed according to [30] with a calcium transfection kit purchased from Clontech (Takara Bio, Kusatsu, Japan, #631312). For neuronal dendritic spines morphology analysis pLV-eGFP (36083, Addgene, Watertown, MA, USA), plasmids were used. At DIV 14–16, the cells were fixed with 4% paraformaldehyde in PBS for 10–15 min at room temperature and mounted onto glass slides for imaging. For neuronal dendritic spines morphology analysis, z-stacks of neuronal dendritic spines were captured with a confocal microscope (Leica TCS SP8) using 100× objective lens (UPlanSApo, Olympus, Japan). Optical sections were captured at a resolution of 0.022 µm/pixel, and the size of each optical section was 2048 × 2048 pixels (Figure 5).

To detected neuronal endoplasmic reticulum, at DIV7 neurons were transfected with DsRed-ER plasmid (Clontech, Mountain View, CA, USA #632409). At DIV 16–17, live cell imaging was performed using a confocal microscope (ThorLabs, Blairstown, NJ, USA) using 100× objective lens (UPlanSApo, Olympus, Japan). Optical sections were captured at a resolution of 0.0185 µm/pixel, and the size of each optical section was 2048 × 2048 pixels. The samples were imaged in a glass imaging camera filled with aCSF, pre-heated to 37 °C (Figure 4, Supplementary Figure S9).

To detect dendritic spines from brain slices of line M mice, Thy1-GFP was used as in details described in [30]. Briefly, slices with a thickness of 40–50 µm were prepared. The slices were incubated for 16 h with primary monoclonal antibodies targeting GFP proteins (1:200 dilution, Invitrogen, Waltham, MA, USA, Cat. No. MA5–32977). Afterwards, the samples were stained with secondary antibodies: Alexa-Fluor 594 (2 drops per mL, anti-mouse, Invitrogen, Cat. No. R37121) and Alexa-Fluor 488 (2 drops per mL, anti-rabbit, Invitrogen, Cat. No. R37116) for 2 h (Figure 5).

To detect IP31 cluster in brain slices from 6-mouth 5xFAD mice, expansion microscopy was used. Slices with a thickness of 40–50 µm were made. To enhance the staining, the HCl-antigen retrieval method was used, which is necessary for image analysis using expansion microscopy, since sample expansion leads to a decrease in the concentration of fluorophores and, as a result, the brightness of the resulting signal. Incubation with primary monoclonal antibodies to polyclonal IP3R1 (1:300 dilution, Abcam, cat. no. ab5804) was carried out for 16 h, after which there was staining with secondary antibodies Alexa-Fluor 488 (diluted two drops per mL, anti-rabbit, Invitrogen, Cat. No. R37116) for 2 h. Then, the section was expanded in accordance with the protocol of expansion microscopy [29] to enlarge the sample and visualized using confocal microscopy (0.072 µm/pixel, 2048 × 2048 pixels) (Supplementary Figure S8).

For RLTV method comparison, experimentally acquired PSFs were obtained from 200 nm fluorescent sphere capturing with the optical system parameters matching the imaging configurations. The PSFs were calculated using simple-PSF software (https://github.com/gerasimenkoab/simple-PSF, accessed on 24 August 2025). Four types of PSF were used, including the following: the system PSFs of the high-resolution system (Abberior Facility Line, Abberior Instruments GmbH, Germany) in the confocal laser scanning mode (Figure 2), the system PSFs of the confocal microscope (Thorlabs, ×60, NA of 0.8 water objective) for the test of the STIM1 clusters from HEK293T and the spines from neuronal culture (Figure 3), the system PSFs of the confocal microscope (Leica TCS SP8, ×100, NA of 1.4 oil objective) for the test of the spines from neuronal culture and brain slices (Figure 5), and growth cones from neuronal culture (Figure 4); and the system PSFs of the confocal microscope (Thorlabs, ×100, NA of 1.42 oil objective) for the test of the live endoplasmic reticulum from neuronal culture (Figure 4).

The result of bead extraction is presented in ‘NeuroDecon-data.zip’ Supplementary Materials (see ‘spheres_images’ folder).

### 4.5. Image Quality Assessment

In all experiments, the following equations were used to image quality metrics:

$$SSIM=\frac{\left(2\mu_{x}\mu_{y}\;+\;C_{1})(2\sigma_{xy}\;+\;C_{2}\right)}{\left({\mu_{x}}^{2}\;+\;{\mu_{y}}^{2}\;+\;C_{1})({\sigma_{x}}^{2}\;+\;{\sigma_{y}}^{2}\;+\;C_{2}\right)}$$

where for non-negative image signals i, j, µ_i_ is the mean intensity of i; σ_i_ is standard deviation of i; σ_ij_ is the correlation coefficient for I; j, and C_n_ are constants introduced to prevent divergence when the aforementioned values are close to zero, in accordance with the literature data [9] (Figure 1 and Figure 5).

$$RMSE=\sqrt{\frac{\sum_{x,y}\;{{(I}_{1}\left(x,y\right)-I_{2}\left(x,y\right))}^{2}}{n}}$$

where for non-negative image signals i, j, I_i_ is the intensity of a pixel with given x, y coordinates, and *n* is the number of pixels analyzed (Figure 4).

$$Pearson\;correlation=\frac{\sum_{x,y}\left(I_{1}\left(x,y\right)-\bar{I_{1}})(I_{2}\left(x,y\right)-\bar{I_{2}}\right)}{\sqrt{\sum_{x,y}\left(I_{1}\left(x,y\right)-\bar{I_{1}}\right)}\sqrt{\sum_{x,y}{(I}_{2}\left(x,y\right)-\bar{I_{2}})}}$$

where for a non-negative image signal i, I_i_ is the intensity of a pixel with given x, y coordinates, and $\bar{I}i$ is the mean value of said intensity over a given area (Figure 4).

SNR and resolution were estimated with ImDecorr plugin [31] (Figure 2, Figure 3, Figure 4 and Figure 5, Supplementary Figures S4–S6).

### 4.6. Statistical Analysis

Statistical data analysis was performed using GraphPad Prism software (version 10.5.0). To determine the distribution for a metric, the Shapiro–Wilk test or Kolmogorov–Smirnov test was calculated depending on the number of data points available (<10 for Shapiro–Wilk test and ≥10 for Kolmogorov–Smirnov test). If the distribution was not normal, the Kruskal–Wallis test or the Mann–Whitney test was used to evaluate distinctions between groups. For small-size samples (*n* < 30), Conover–Iman post hoc test was used for multiple pairwise comparisons. For *n* > 30, Dunn’s post hoc test was used. If the obtained results followed a normal distribution, the equality of variances was confirmed by assessing the data using the Bartlett test. Subsequently, a standard one-way analysis of variance (ANOVA) was conducted, followed by the utilization of Tukey’s test for the post hoc analysis. If the equality of variances was not confirmed, Welch and Brown-Forsythe ANOVA was conducted, followed by the utilization of Dunnett’s T3 for the post hoc analysis. The statistical tests used, along with *p*-values, are presented in Supplementary Table S3 (Figure 1, Figure 2, Figure 3, Figure 4 and Figure 5, Supplementary Figures S4–S6).

## Data Availability

The data that support the findings of this study are included in Figure 2, Figure 3, Figure 4 and Figure 5, Supplementary Figures S4 and S5. Other datasets (training data for deep learning) are available at https://zenodo.org/records/15127791, accessed on 24 August 2025 or in Supplementary Files (Neuro-Decon-data.zip). NeuroDecon is available at https://github.com/Biomed-imaging-lab/NeuroDecon, accessed on 24 August 2025 or in Supplementary Files (Neuro-Decon-code.zip). RCAN software was installed from https://github.com/AiviaCommunity/3D-RCAN, accessed on 24 August 2025.

## Supplementary Materials

The following supporting information can be downloaded at: https://www.mdpi.com/article/10.3390/ijms26188770/s1.

## Abbreviations

The following abbreviations are used in this manuscript:

PSF	Point Spread Function
RLN	Richardson–Lucy Network
RLTV	Richardson–Lucy Total Variation
PSNR	Peak Signal-to-Noise Ratio
SSIM	Structure Similarity
STED	Stimulated Emission Depletion Microscopy
ExM	Expansion Microscopy
ER	Endoplasmic Reticulum
ASM	Angular Second Moment
ANOVA	Analysis of Variance
