# Supplementary material for: NeuroDecon: A Neural Network-Based Method for Three-Dimensional Deconvolution of Fluorescent Microscopic Images
Source: Int J Mol Sci. 2025 Sep 9;26(18):8770. doi: 10.3390/ijms26188770 (PMC12469540; doi:10.3390/ijms26188770)
Supplement: Supplementary file 1 [file ijms-26-08770-s001.zip › Supplementary_materials.pdf]

Supplementary Materials for
NeuroDecon: a neural network-based method for three-dimensional deconvolution of fluorescent microscopic images

This file includes:
Supplementary Text
Supplementary Figures S1 to S13
Supplementary Tables S1 to S3

1. Synthetic tubes generation

For generation of synthetic tubes images, here is provided step-by-step algorithm:

1) Generating a directing vector in spherical coordinates. For this purpose, directing vector can be described by two angels: $\varphi \in U(-\pi, \pi)$ – rotate angel in OXY plane into Cartesian systems, $\theta \in U(-\frac{\pi}{2}, \frac{\pi}{2})$ – angel between directing vector and OZ. Note that U denotes as uniform continuous distribution.

2) Translation of the generated vector into Cartesian systems:

$$\begin{cases} x_m = \sin(\theta)\cos(\varphi) \\ y_m = \sin(\theta)\sin(\varphi) \\ z_m = \cos(\theta) \end{cases}$$

where (x_m, y_m, z_m) – coordinates of the direction vector of the line.

3) Generation of a point which will be passed by a tube. This point can be generating by choosing coordinates by uniform discrete distribution of image points. Let us denote the coordinates as (x_c, y_c, z_c).

4) Generation of points belonging to a line. The points belonging to the line can be calculated by the following system:

$$\begin{cases} x = x_c + \mu x_m \\ y = y_c + \mu y_m \\ z = z_c + \mu z_m \end{cases}$$

where μ –real number.

To generate points belonging to the line, it is necessary to calculate the minimum and maximum values of the coefficient μ. It can be done in this way: put coordinates (x, y, z) equal to zero and to sizes of the three-dimensional image along the corresponding axes. Then, deriving from these equalities the values of μ and calculating them. After this step 6 different values of μ can be obtained. So, let denotes the minimum non-negative values from them as μ_M and the maximum non-positive μ_m. These two new variables are describing interval of values [μ_m, μ_M], where μ_m, μ_M – minimum and maximum values of μ for finding points of image which are belonging to a line.

5) Generating a straight tube. Using defined interval [μ_m, μ_M], it is possible to take some number of intermediate equidistant values μ from the interval and calculate with them the coordinates of the points using formulas from step 4. Rounded to nearest integer results of that system scan be taken as points of image

corresponding to the line. Heuristically, the number of values μ can be estimated as $[\sqrt{X_{size}^2 + Y_{size}^2 + Z_{size}^2}]$, where $X_{size}, Y_{size}, Z_{size}$ – volumetric image sizes along the axes, and [.] – rounding to integer operation.

6) Convolution the obtained image of a straight line with some generated sphere using the sphere generation algorithm. This convolution allows to generate straight lines of different thicknesses, shapes and brightness.

2. Data generation procedure

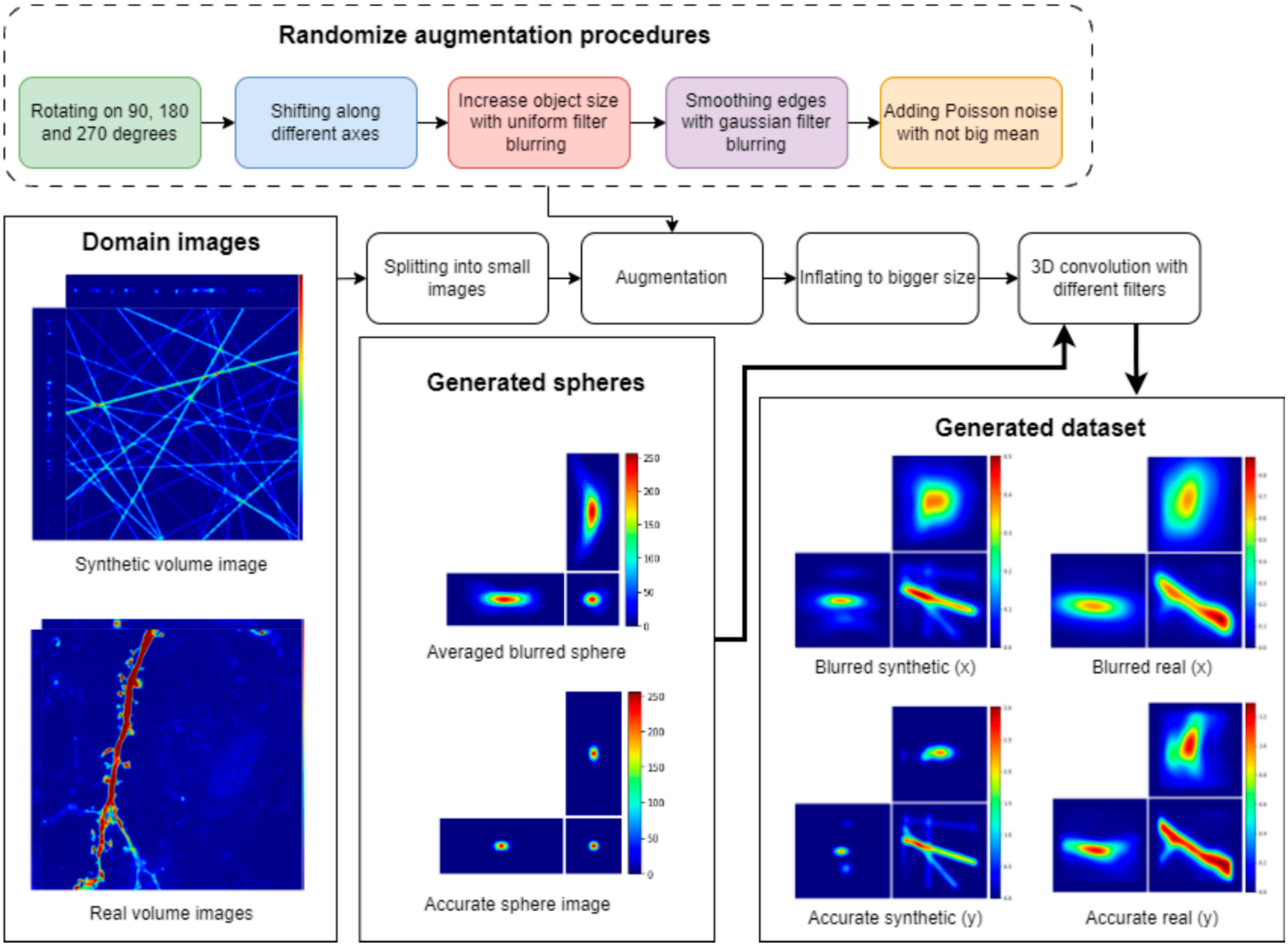

Supplementary Figure S1. The dataset generation pipeline: domain images splitting on some size, when augmented to create some new patches, inflating to some bigger size and, finally, convoluted with blurred and accurate spheres

3. *NeuroDecon testing metrics on synthetic spheres and tubes and on real spheres*

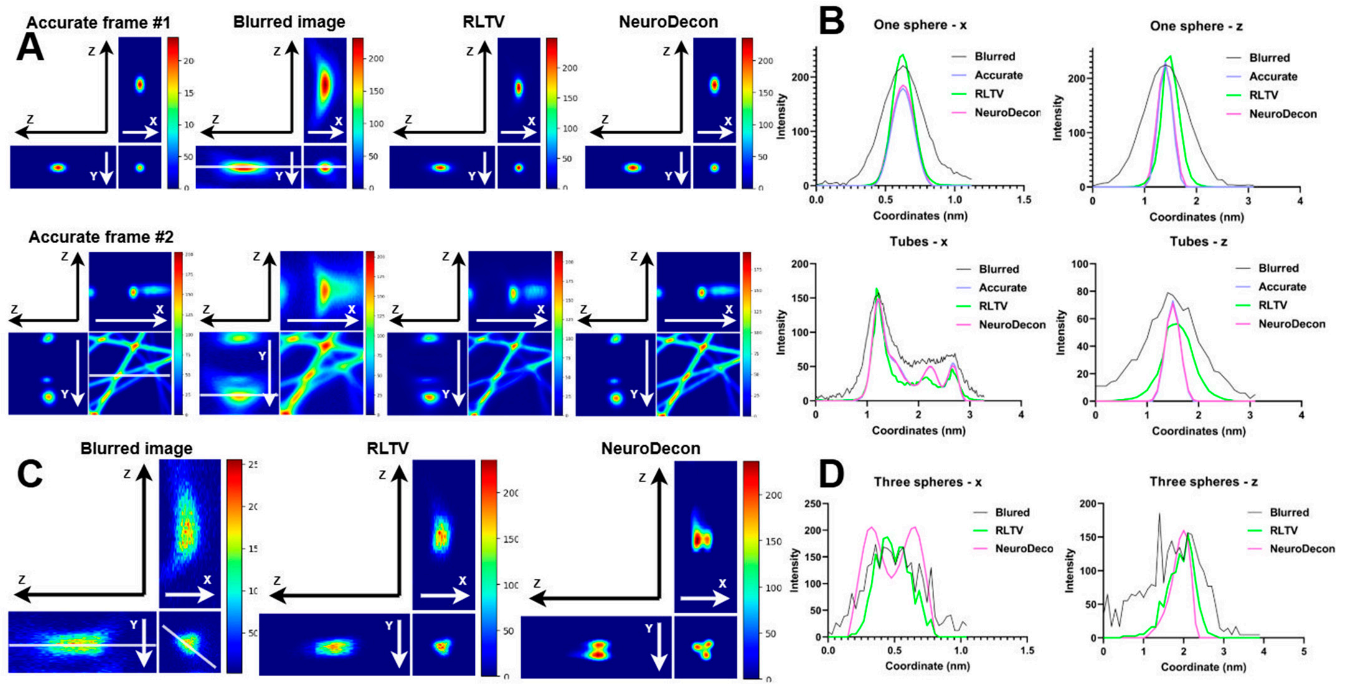

Supplementary Figure S2. NeuroDecon testing metrics on synthetic and real data with tubes and spheres. (A) Representative zoomed frames and (B) intensity plots along the line from images on the panel (A); (C) A representative confocal microscopy image of 3 clumped fluorescent spheres before and after NeuroDecon processing showing unachievable with RLTV deconvolution objects separation and resolution enhancement; (D) Intensity plots along the line in (C). Source data for all experiments are provided with this manuscript and summary statistics are provided in Supplementary Table S3.

4. *NeuroDecon testing on synthetic data with different noise levels*

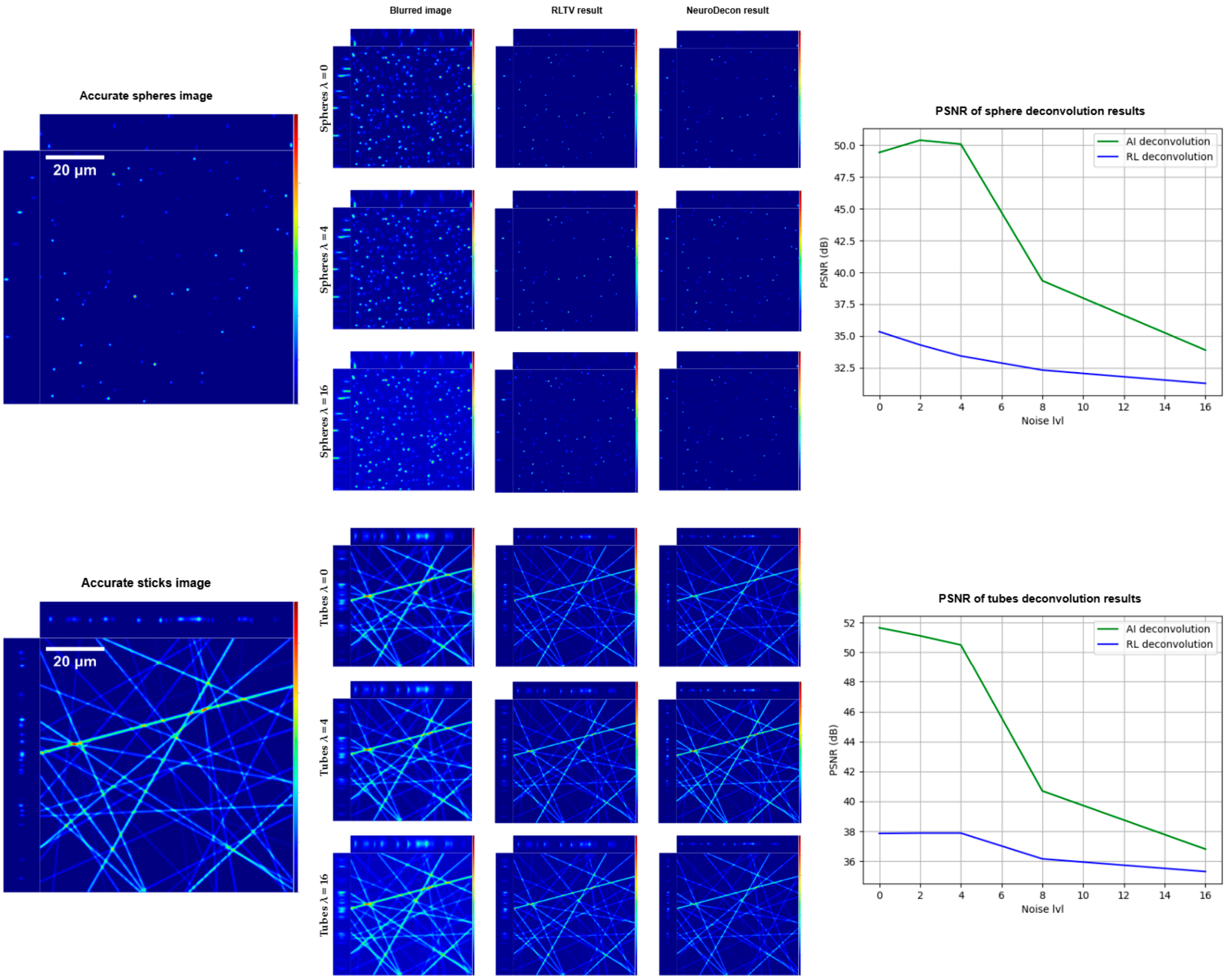

Supplementary Figure S3. NeuroDecon testing on synthetic noisy data. Demonstration of the noise tolerance of the NeuroDecon method when working with noisy synthetic images in comparison with the RLTV method.

5. NeuroDecon performance on different confocal images of tubes

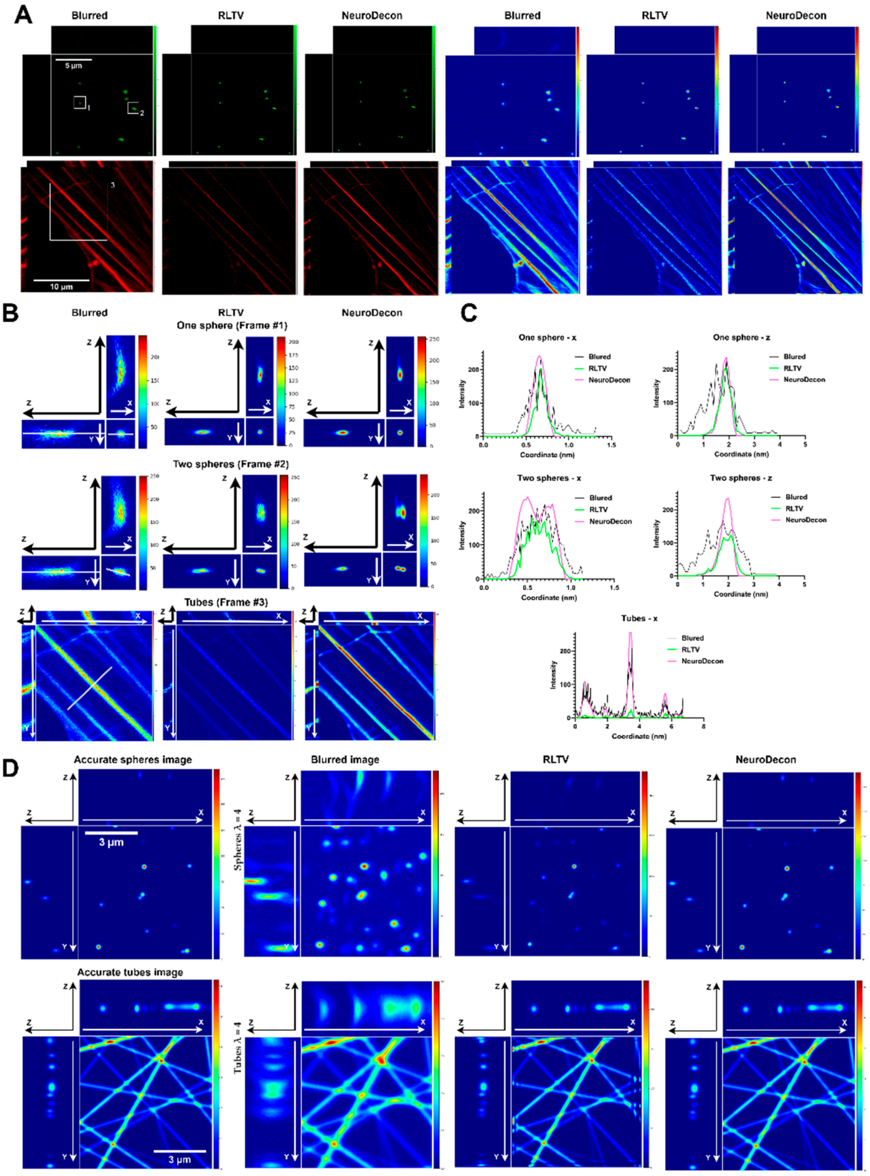

Supplementary Figure S4. NeuroDecon performance on different tubes and confocal images**:** (**A**) Demonstration of NeuroDecon method working with real images of tubes and spheres in comparison with the RLTV method: in original color map and Jet color map; (**B**) Parts of real images of spheres and tubes presented as triples Original image, deconvolved by RL, deconvolved by NeuroDecon; (**C**) Plots of intensities along selected translucent white lines for each corresponding part of the original images; (**D**) Example images of synthetic spheres and tubes that participated in the interval estimation of PSNR and SSIM values after deconvolution by two different methods.

6. SNR and Resolution values across different planes from "NeuroDecon resolution enhancement of confocal images is comparable with STED super resolution microscopy"

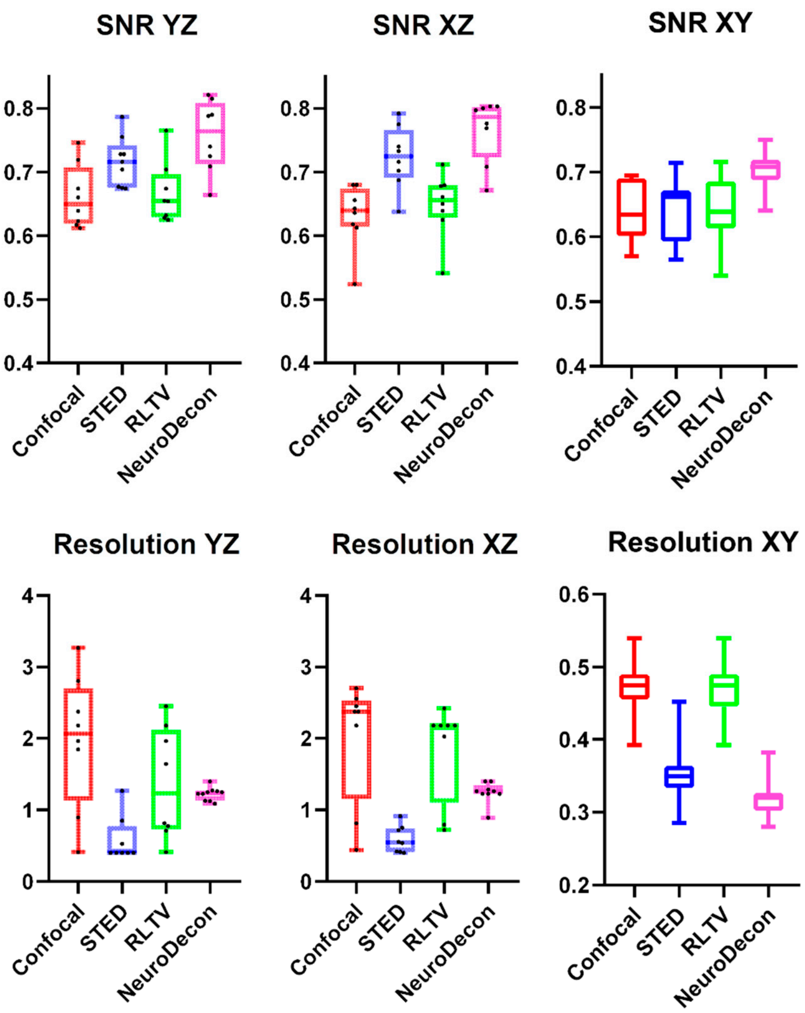

Supplementary Figure S5. Statistical comparisons of confocal images, STED images, RLTV deconvolution, and Neu-roDecon for signal-to-noise ratio (SNR) and resolution metrics across the XZ, YZ, and XY planes, n=8 for all data regarding XZ and YZ planes and n = 14 for all data regarding XY plane. Source data for all experiments are provided with this manuscript and summary statistics are provided in Supplementary Table S3.

7. Average NeuroDecon neuronal network training results

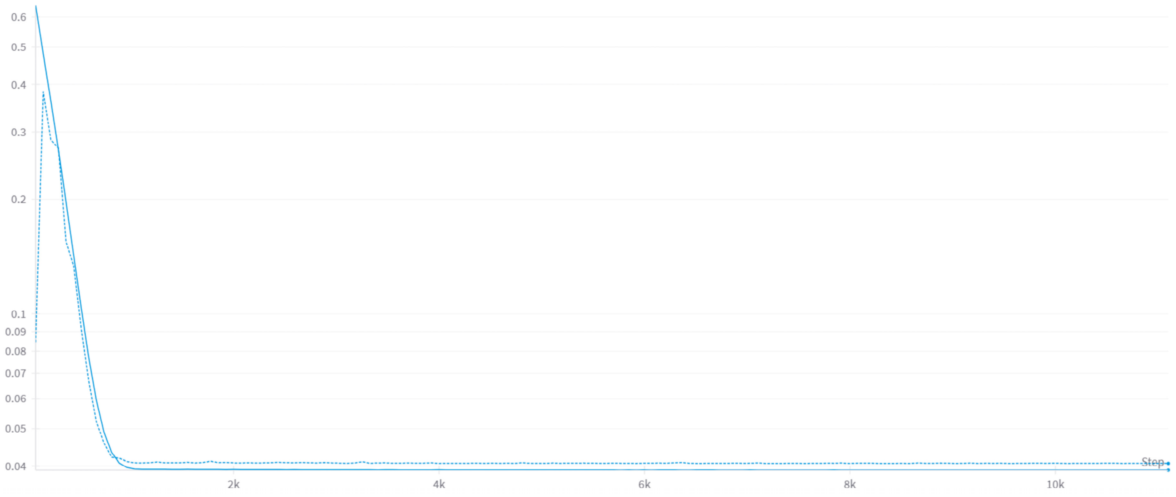

Supplementary Figure S6. The function of the error graph from the step of updating the weights: blue plain line – loss on training dataset, blue stripped line – loss on validation dataset. The error in both validation and training samples converge to a constant long before the end of training.

8. Residual block architecture

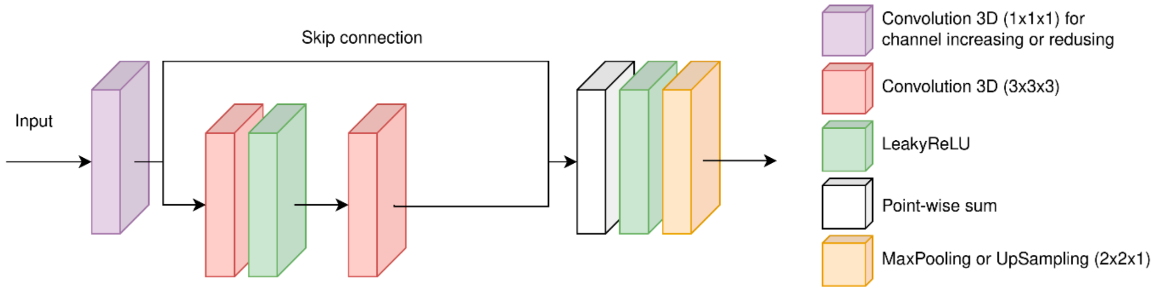

Supplementary Figure S7. Residual block architecture. On the left: one residual block architecture. On the right: description of layers.

9. Demonstration of NeuroDecon for enhanced resolution and noise reduction in 3D-imaging on fluorescent spheres.

To demonstrate more clearly the performance of NeuroDecon, we have conducted a series of measurement experiments on 200nm fluorescent spheres images (Supplementary Figure S6a). The analysis of intensity profiles (Supplementary Figure S6b) revealed significant improvement of axial resolution by NeuroDecon method. As is evident in the representative axial projections (Supplementary Figure S6c), NeuroDecon also provided great noise reduction. Singular-sphere projection in Supplementary Figure S6d highlight that the image quality enhancement is isotropic, and that is further proved by statistical analysis of resolution and SNR provided in Supplementary Figure S6e, as NeuroDecon showed strong SNR enhancement compared to raw confocal images, and has provided resolution close to STED microscopy. Statistical analysis details are provided in Supplementary Table S3.

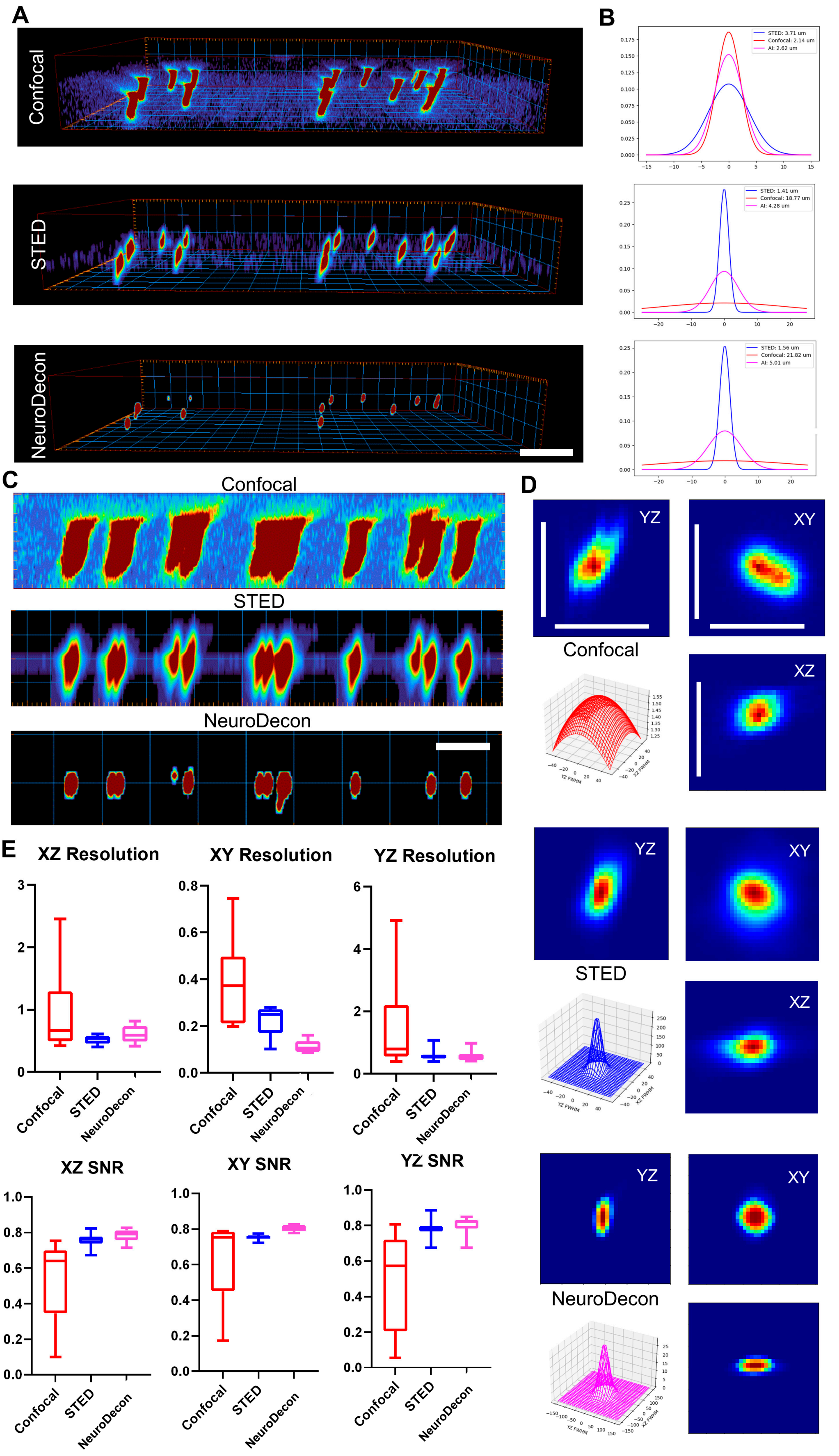
A
Confocal
STED
NeuroDecon
B
STED: 3.71 um
Confocal: 2.14 um
AI: 2.62 um
STED: 1.41 um
Confocal: 18.77 um
AI: 4.28 um
STED: 1.56 um
Confocal: 21.82 um
AI: 5.01 um
C
Confocal
STED
NeuroDecon
D
YZ
XY
XZ
Confocal
STED
NeuroDecon
E
XZ Resolution
XY Resolution
YZ Resolution
XZ SNR
XY SNR
YZ SNR
Confocal
STED
NeuroDecon

Supplementary Figure S8. Demonstration of NeuroDecon for enhanced resolution and noise reduction in 3D-imaging on fluorescent spheres: **(a)** A 3D-reconstruction of fluorescent spheres from raw confocal images, STED images and NeuroDecon deconvolution results. Scale bar corresponds to 10 μm; (**b)** Intensity profile plots in XY, XZ and YZ planes and according FWHM; (**c)** Axial projections of raw confocal images, STED images and NeuroDecon deconvolution results. Scale bar corresponds to 10 μm; (**d)** Singular sphere projections on XY, XZ and YZ planes and 2d-profiles for XZ/YZ planes for raw confocal images, STED images and NeuroDecon deconvolution results. Scale bars correspond to 1 μm for XY plane, 5 μm for XZ and YZ planes; (**e)** Statistical comparisons of confocal images, STED images and NeuroDecon for signal-to-noise ratio (SNR) and resolution metrics across the XZ, YZ, and XY planes.

10. ***Improving resolution and reducing noise in ExM with NeuroDecon for images with high noise levels.***

The inclusion of Poisson noise in the training images dataset has shown to allow for signal restoration even from images with a significant amount of noise. Images of IP3R protein clusters acquired from expanded brain slices (Supplementary Figure S7 a,b) have been chosen as an example of such data. NeuroDecon deconvolution method has shown a great capacity for image restoration even in the presence of noise, as is evident in the representative pictures (Supplementary Figure S7c). The analysis of intensity profiles (Supplementary Figure S7d) revealed significant improvement of lateral and axial resolution by NeuroDecon method, and further statistical analysis revealed a significantly higher SNR provided by NeuroDecon in comparison to RLTV deconvolution. However, overall statistical analysis showed increased resolution after NeuroDecon and RLTV deconvolution, which could be interpreted as a side effect of noise reduction. Statistical analysis details are provided in Supplementary Table S3.

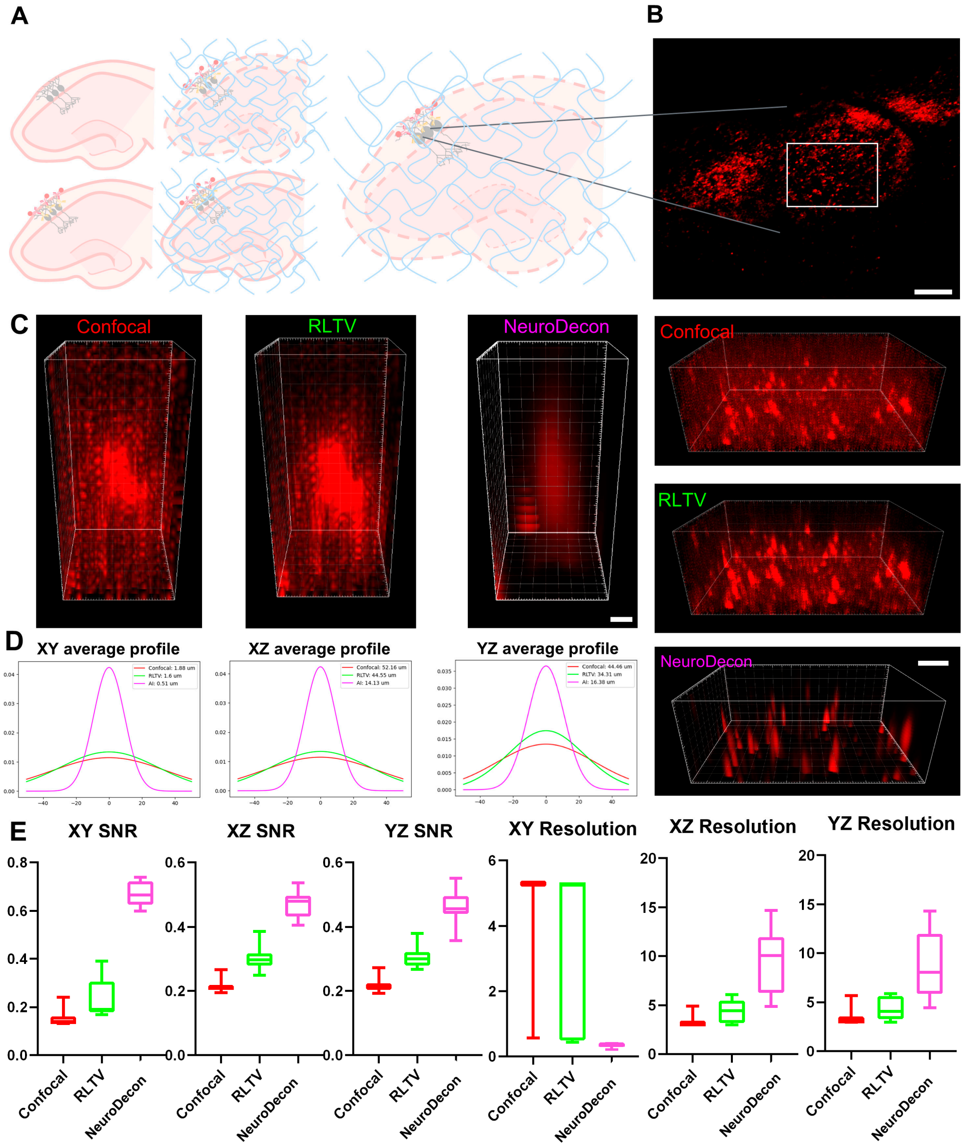

Supplementary Figure S9. Improving resolution and reducing noise in ExM with NeuroDecon for images with high noise levels**:** (**a**) a schematic depiction of ExM sample preparation procedure; (**b**) A raw confocal image of IP3R clusters in antibody-stained brain slices. Scale bar corresponds to 10 µm; **(c)** On the right, from top to bottom, 3D-reconstructed clusters from the boxed region in B of an unprocessed image, the results of RLTV and NeuroDecon, on the right, 3D-reconstructions of singular clusters. Scale bar corresponds to 0.5 µm for the images on the left and 2 µm for the images on the right; (**d**) Gauss-fitted statistical intensity plots of clusters with corresponding FWHM across the XZ, YZ, and XY planes; (**e**) Statistical comparisons of confocal images, RLTV deconvolution, and NeuroDecon for signal-to-noise ratio (SNR) and resolution metrics across the XZ, YZ, and XY planes.

11. NeuroDecon improves live- and fixed- cell confocal image quality to reveal intricate organelle structure

The usage of global metrics, such as global SNR, resolution or global SSIM on images with a distinct area of interest (such as an organelle within a cell, as in Supplementary Figure 8a) requires maps of such metrics assessed locally to avoid drawing inconsistent or incorrect conclusions. Therefore, we present SNR and normalized cutoff frequency maps (Supplementary Figure 8b) that suggest that the globally-assessed parameters (Supplementary Figure 8c) do align with the local estimates in the region of interest. Additionally, we have measured SSIM for areas with thin and thick tubules (Supplementary Figure 8d), and the results have shown that areas with narrower tubules do not disproportionately affect SSIM, and that similar tendencies of image restoration are present in all areas of the tubular network. Statistical analysis details are provided in Supplementary Table S3.

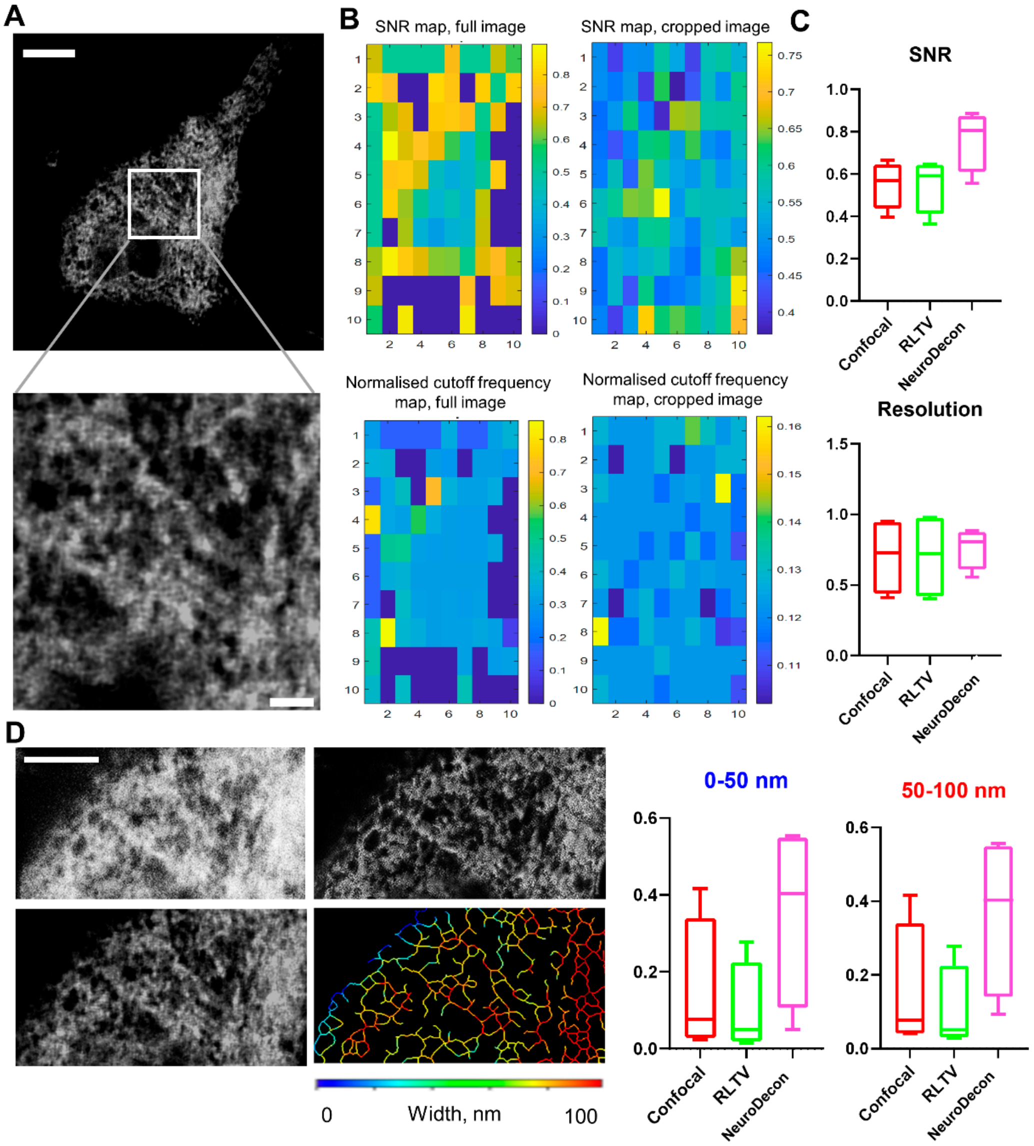

Supplementary Figure S10. NeuroDecon improves live- and fixed- cell confocal image quality to reveal intricate organelle structure: (**a**) A confocal image of neuronal ER treated with NeuroDecon and a highlighted region of interest within the picture. Scale bars correspond to 5 µm for the full image and to 1 µm for the cropped image; (**b**) SNR and normalized cutoff frequency maps for images in (a); (**c**) Statistical comparisons of confocal images, RLTV deconvolution, and NeuroDecon for signal-to-noise ratio (SNR) and resolution metrics across the XY plane; (**d**) A representative confocal image of unprocessed neuronal ER (top left), RLTV results (top right) NeuroDecon results (bottom left) and skeletonized ER (bottom right), and statistical comparison of SSIM for areas with narrower tubules (0-50 nm) and wider tubules (50-100 nm). Scale bar corresponds to 5 µm.

To ensure the minimal presence of artifacts in NeuroDecon-produced images, an additional experiment of actin filament image restoration in growth cones has been conducted. As is evident in the representative images (Figure S9a), NeuroDecon has revealed the fine structure of the actin filament ensemble of the growth cone in a more consistent way throughout the image than RLTV deconvolution method. Quantitative analysis has con-firmed that the results of NeuroDecon deconvolution have a higher root-mean-square-error between them and the confocal images than the results of RLTV deconvolution (Figure S9b), while simultaneously remaining strongly correlated to them (Figure S9b), suggesting little to no artifacts. The error maps of the resulting images compared to the raw image (Figure S9c) highlight the uniformity of noise reduction by NeuroDecon throughout the image and explicitly show background noise reduction by NeuroDecon. Statistical analysis details are provided in Supplementary Table S3.

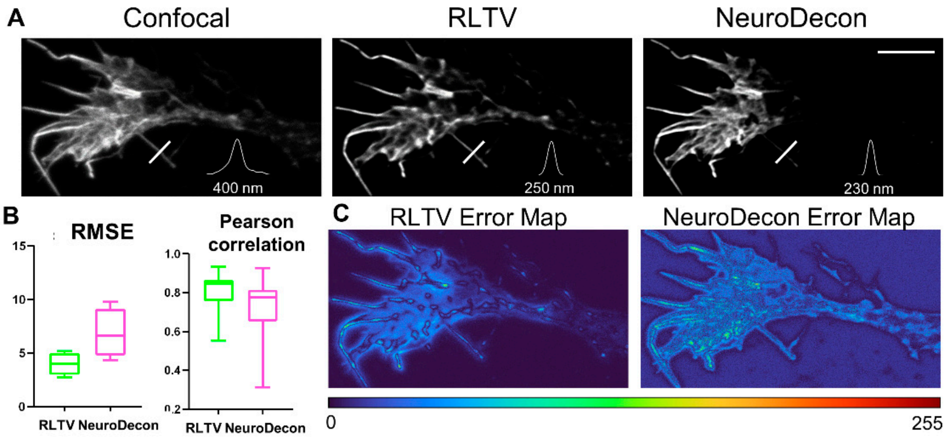

Supplementary Figure 11. (**a**) Neuronal growth cone images, from left to right, unprocessed confocal image, RLTV deconvolution, NeuroDecon deconvolution. Scale bar corresponds to 5 μm; (**b**) Root-mean-square-error (RMSE) and Pearson correlation of the results of RLTV and NeuroDecon deconvolution methods and unprocessed confocal images, n=14; (**c**) RMSE Error maps of the results of RLTV and NeuroDecon deconvolution methods and unprocessed confocal images.

12. Enabling efficient neuronal dendritic spine morphology analysis with NeuroDecon

Dendritic spines are responsible for the majority of excitatory synaptic contacts, and suffer shape alterations in many neurodevelopmental and neurodegenerative disorders. Although it is possible to assess the morphology of dendritic spines with manual methods, automatic spine segmentation is preferable for analysis, as it is less subjective and more consistent. However, excessive noise in confocal spine images can misrepresent the details of spine shapes, or fully obscure the thin parts, such as spine necks, which hinders automatic analysis and makes it less reliable. This effect is most noticeable in in vivo spine analysis, i.e. in images of brain slices, where the spines that lie closer to the dendrite have thin, hardly distinguishable necks. NeuroDecon has shown to be able to combat this issue. We have applied RLTV and NeuroDecon deconvolution methods to images of hippocampal dendritic spines in vitro, in vivo and after expansion microscopy. The images were then used to reconstruct 3D meshes of dendritic spines using SpineTool software (Figure S10a). Quantitative analysis revealed that using RLTV deconvolution allowed to increase the number of successfully segmented and reconstructed spines for images with little to no background noise, such as in vitro spines and ExM spines (Figure S10b, c), yet has failed to yield similar results for noisy images, as can be seen by the disappearance of thin-neck in vivo spines (Figure S10d). Applying the NeuroDecon method, however, allowed for enhanced spine segmentation with little dependence on image noise levels (Figure S10b-d). Applying deconvolution methods to dendritic spine images also improves the absolute accuracy of spine volume estimation (Figure S10b-d), as the microscope aberration contributes to their perceived enlargement. In the case of NeuroDecon treatment of in vivo spine images, the successfully-segmented spines with large heads and thin necks have contributed to overall spine volume enlargement (Figure S10d), as also supported by greater variance in spine volumes, which suggests that analytical deconvolution may interfere with drawing correct biological conclusions from collected spine segmentation data, acting as an unaccounted-for sampling bias. All statistical analysis details are provided in Supplementary Table S3. These results demonstrate the accuracy of spine analysis results acquired with the application of the NeuroDecon deconvolution method.

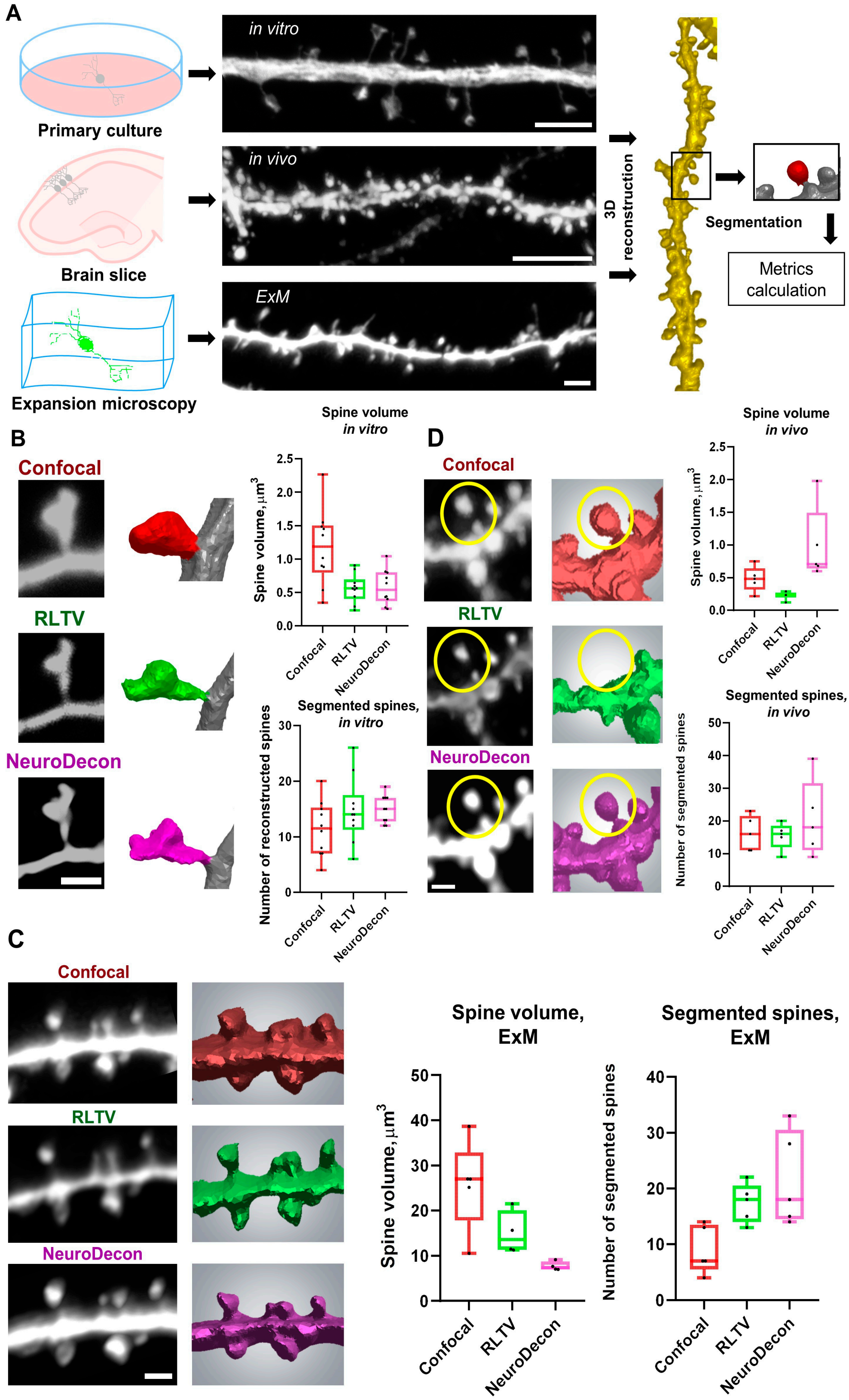
A
Primary culture
Brain slice
Expansion microscopy
in vitro
in vivo
ExM
3D reconstruction
Segmentation
Metrics calculation
B
Confocal
RLTV
NeuroDecon
Spine volume in vitro
Spine volume, μm3
Segmented spines, in vitro
Number of reconstructed spines
D
Spine volume in vivo
Segmented spines, in vivo
Number of segmented spines
C
Spine volume, ExM
Segmented spines, ExM

Supplementary Figure S12. Enabling efficient dendritic spine morphology analysis with NeuroDecon: (**a**) A schematic depiction of spine segmentation data collection. Scale bars correspond to 5 µm each; (**b**) From left to right, representative confocal images of an in vitro dendritic spine, the results of its 3D-reconstruction with SpineTool and statistical analysis of in vitro spine segmentation and volume evaluation results ($n \geq 10$ spines for $n=9$ images). Scale bar corresponds to 1 µm; (**c**) From left to right, representative confocal images of ExM dendritic spines and the results of their 3D-reconstruction with SpineTool. Statistical analysis of ExM spine segmentation and volume evaluation results ($n \geq 10$ spines for $n=4$ images). Scale bar corresponds to 2 µm; (**d**) From left to right, representative confocal images of in vivo dendritic spines, the results of their 3D-reconstruction with SpineTool, and statistical analysis of in vivo spine segmentation and volume evaluation results ($n \geq 10$ spines for $n=5$ images). Scale bar corresponds to 1 µm. Source data for all experiments are provided with this manuscript and summary statistics are provided in Supplementary Table S3.

13. **Resolution and SNR values along different axis from** "NeuroDecon and similar existing deep learning method for image restoration"

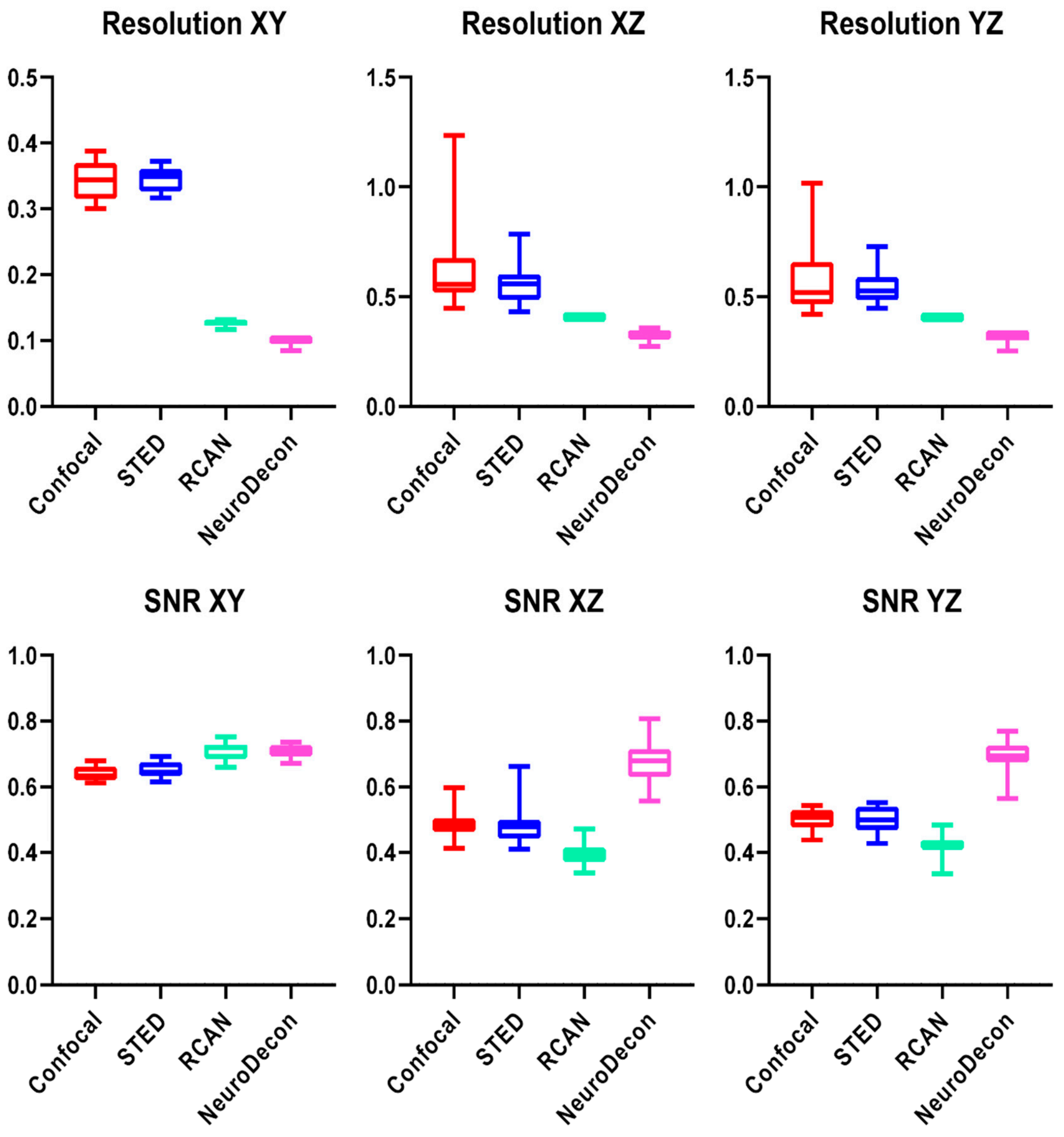

Supplementary Figure S13. Statistical comparisons of confocal images, STED, RCAN and NeuroDecon for signal-to-noise ratio (SNR) and resolution metrics across the XZ, YZ, and XY planes, n=20 for all data

14. Supplementary Table S1. Dataset characteristics.

Dataset characteristics' set name	Images based on	Splitting size, pixels	Was augmented?	Augmentation data part	Final images size, pixels
Synthetic dataset	Synthetic generated images of spheres and tubes	(30; 150; 150)	+	75%	(36; 160; 160)
Neurons dataset	Truth-ground preprocessed images of neurons			~66%	
STIM dataset+	Truth-ground preprocessed images of neurons, endoplasmatic reticulum,			50%	
IP3R dataset+	Truth-ground preprocessed images of neurons, endoplasmatic reticulum			50%	
Growth cones dataset+	Growth cones, endoplasmatic reticulum, truth-ground preprocessed images of neurons			50%	

15. Supplementary Table 2. Training parameters.

Experiment name	Dataset name	Train/test split	Additional Poisson noise level	Epochs	Alpha regularize value
Synthetic testing	Synthetic dataset	4:1	$\varepsilon \in U([0,1,2,3,4,5,6])$	200	0
Real testing on primitives					
Spine cultures	Neurons dataset+				4.8
Astrocytes					
Expansion STIM	STIM dataset+				0.7
Live ER	Growth cones dataset+				0.7
Growth cones	Growth cones dataset+				0
IP3R clusters	IP3R dataset+				0

16. Supplementary Table S3.

The results of statistical analysis are presented as p-values at the intersections of the appropriate groups in the bottom left part of the table. The data is presented as mean [mean-SEM, mean+SEM] for data that follows a normal distribution and as median [Q1 Q3] for data that does not follow a normal distribution on the main diagonal at the intersection of the group with itself. The statistical tests used are presented at the intersections of the appropriate groups in the top right part of each table section.

**Figure 1**	**RLTV**	**NeuroDecon**
**PSNR Spheres**		
RLTV	36.48 [36.1754 36.7846]	Unpaired t test
NeuroDecon	<0,0001	50.23 [49.7581 50.7019]
**SSIM Spheres**		
RLTV	0.03622 [0.03376 0.03868]	Unpaired t test
NeuroDecon	<0,0001	0.93690.9273960.946404
**PSNR Tubules**		
RLTV	30.54 [30.2429 30.8371]	Unpaired t test
NeuroDecon	<0,0001	45.15 [43.082 47.218]
**SSIM Tubules**		
RLTV	0.09519 [0.0832 0.10718]	Unpaired t test
NeuroDecon	0.0003	0.4814 [0.39783 0.56497]

**Figure 2**	**STED**	**Confocal**	**RLTV**	**NeuroDecon**
**Resolution XY**				
STED	0.3499 [0.3334 0.3635]	Dunn's	Dunn's	Dunn's
Confocal	<0,0001	0.4745 [0.455 0.49]	Dunn's	Dunn's
RLTV	<0,0001	>0,9999	0.4745 [0.4453 0.49]	Dunn's
NeuroDecon	0.1413	<0,0001	<0,0001	0.3197 [0.3025 0.3267]
**Resolution YZ**				
STED	0.4026 [0.4016 0.7713]	Dunn's	Dunn's	Dunn's
Confocal	0.0018	2.069 [1.133 2.693]	Dunn's	Dunn's
RLTV	0.1171	>0,9999	1.228 [0.728 2.123]	Dunn's
NeuroDecon	0.0854	>0,9999	>0,9999	1.237 [1.128 1.265]
**Resolution XZ**				
STED	0.5461 [0.4161 0.7429]	Dunn's	Dunn's	Dunn's
Confocal	0.0008	2.373 [1.155 2.526]	Dunn's	Dunn's
RLTV	0.0079	>0,9999	2.178 [1.102 2.178]	Dunn's
NeuroDecon	0.105	0.7362	>0,9999	1.263 [1.225 1.342]
**SNR YZ**				
STED	0.716 [0.67733 0.75467]	Tukey's	Tukey's	Tukey's
Confocal	0.1098	0.6613 [0.61199 0.71061]	Tukey's	Tukey's
RLTV	0.1774	0.9947	0.6671 [0.61952 0.71468]	Tukey's
NeuroDecon	0.3226	0.0023	0.0044	0.7565 [0.70073 0.81227]
**SNR XY**				

STED	0.661 [0.5925 0.6715]	Dunn's	Dunn's	Dunn's
Confocal	>0,9999	0.634 [0.6015 0.69]	Dunn's	Dunn's
RLTV	>0,9999	>0,9999	0.6386 [0.613 0.685]	Dunn's
NeuroDecon	<0,0001	<0,0001	<0,0001	0.708 [0.688 0.7185]
**SNR XZ**				
STED	0.7245 [0.6908 0.7663]	Dunn's	Dunn's	Dunn's
Confocal	0.0461	0.6395 [0.6143 0.674]	Dunn's	Dunn's
RLTV	0.1978	>0,9999	0.6555 [0.6285 0.6788]	Dunn's
NeuroDecon	>0,9999	0.0011	0.0083	0.7865 [0.7233 0.8023]

**Figure 3**	**Confocal**	**RLTV**	**NeuroDecon**
**Resolution XY**			
Confocal	1.299 [1.119 1.745]	Dunn's	Dunn's
RLTV	0.4116	0.8042 [0.7067 1.09]	Dunn's
NeuroDecon	0.0015	0.115	0.467 [0.4377 0.4827]
**Resolution YZ**			
Confocal	10.6 [8.866 12.334]	Tukey's	Tukey's
RLTV	0.2042	7.641 [6.7692 8.5128]	Tukey's
NeuroDecon	0.0013	0.0351	2.808 [2.6973 2.9187]
**Resolution XZ**			
Confocal	8.093 [7.2207 8.9653]	Tukey's	Tukey's
RLTV	0.0042	5.173 [5.0362 5.3098]	Tukey's
NeuroDecon	<0,0001	0.0284	2.885 [2.82154 2.94846]
**SNR YZ**			
Confocal	0.3566 [0.3438 0.3987]	Dunn's	Dunn's
RLTV	0.5464	0.3752 [0.3549 0.4112]	Dunn's
NeuroDecon	0.0005	0.0034	0.4919 [0.425 0.5646]
**SNR XY**			
Confocal	0.4356 [0.41011 0.46109]	Dunn's	Dunn's
RLTV	>0,9999	0.4688 [0.4425 0.4951]	Dunn's
NeuroDecon	0.0116	0.0227	0.5996 [0.597141 0.6021]
**SNR XZ**			
Confocal	0.3683 [0.3593 0.3981]	Dunn's	Dunn's
RLTV	>0,9999	0.3752[0.3611 0.4112]	Dunn's
NeuroDecon	0.0452	0.2011	0.5652 [0.5081 0.5892]

**Figure 4**	**Confocal**	**RLTV**	**NeuroDecon**
**Resolution XY**			
Confocal	0.5469 [0.4684 0.9696]	Dunn's	Dunn's
RLTV	0.005	0.04279 [0.04181 0.04308]	Dunn's
NeuroDecon	0.3481	0.3481	0.1188 [0.1164 0.1657]
**SNR XY**			
Confocal	0.597 [0.57847 0.61553]	Tukey's	Tukey's

RLTV	<0,0001	0.3638 [0.357756 0.369844]	Tukey's
NeuroDecon	<0,0001	0.5944	0.3905 [0.364360.41664]
**Tubule width**			
Confocal	0.07492 [0.070914 0.078926]	Tukey's	Tukey's
RLTV	0.0052	0.0455 [0.040103 0.005397]	Tukey's
NeuroDecon	0.5171	0.0174	0.06827 [0.066196 0.070344]
**Cisternal edge tangent**			
Confocal	2.645 [1.544 3.746]	Tukey's	Tukey's
RLTV	0.0524	9.411 [7.058 11.764]	Tukey's
NeuroDecon	0.9938	0.046	[2.407 1.5519 3.2621]
**Angular Second Moment**			
Confocal	0.2336 [0.12 0.3472]	Tukey's	Tukey's
RLTV	0.7722	0.1275 [0.08838 0.16662]	Tukey's
NeuroDecon	0.0276	0.0096	0.7181 [0.5748 0.8614]
**SSIM**			
Confocal	0.07697 [0.04167 0.3354]	Dunn's	Dunn's
RLTV	>0,9999	0.06193 [0.04274 0.2262]	Dunn's
NeuroDecon	0.35	0.1496	0.4166 [0.1446 0.5515]

Figure 5	STED	Confocal	RCAN	NeuroDecon
**Resolution XY**				
STED	0.3267 [0.3171 0.3499]	Dunn's	Dunn's	Dunn's
Confocal	>0,9999	0.3158 [0.3005 0.345]	Dunn's	Dunn's
RCAN	0.0295	0.0494	0.1242 [0.1171 0.1263]	Dunn's
NeuroDecon	<0,0001	<0,0001	0.0759	0.0949 [0.08465 0.1]
**Resolution YZ**				
STED	0.5254 [0.4838 0.5896]	Dunn's	Dunn's	Dunn's
Confocal	>0,9999	0.5182 [0.4654 0.6569]	Dunn's	Dunn's
RCAN	<0,0001	0.0002	0.4063 [0.4058 0.4071]	Dunn's
NeuroDecon	<0,0001	<0,0001	0.0255	0.313 [0.3102 0.3481]
**Resolution XZ**				
STED	0.5564[0.5198 0.6772]	Dunn's	Dunn's	Dunn's
Confocal	>0,9999	0.5564 [0.5198 0.6772]	Dunn's	Dunn's
RCAN	0.0009	<0,0001	0.4075 [0.4072 0.4078]	Dunn's
NeuroDecon	<0,0001	<0,0001	0.0386	0.3306 [0.3044 0.3481]
**SNR YZ**				
STED	0.4998 [0.491485 0.508115]	Dunn's	Dunn's	Dunn's
Confocal	>0,9999	0.5019 [0.495221 0.508579]	Dunn's	Dunn's
RCAN	0.0007	0.0004	0.4217 [0.414904 0.428496]	Dunn's

NeuroDecon	<0,0001	<0,0001	<0,0001	0.6884 [0.67803 0.69877]
**SNR XY**				
STED	0.6507 [0.644296 0.657104]	Tukey's	Tukey's	Tukey's
Confocal	0.5955	0.6394 [0.633241 0.645559]	Tukey's	Tukey's
RCAN	<0,0001	<0,0001	0.7101 [0.70099 0.71921]	0.7088 [0.704649 0.712951]
NeuroDecon	<0,0001	<0,0001	0.9988	
**SNR XZ**				
STED	0.4781 [0.46592 0.49028]	Dunn's	Dunn's	Dunn's
Confocal	>0,9999	0.4852 [0.47612 0.49428]	Dunn's	Dunn's
RCAN	0.0025	0.0005	0.3955 [0.388088 0.402912]	Dunn's
NeuroDecon	<0,0001	0.0005	<0,0001	0.677 [0.66357 0.69043]

**Supplementary Fig.S6**	**STED**	**Confocal**	**NeuroDecon**
**Resolution XY**			
STED	0.25 [0.1714 0.2718]	Dunn's	Dunn's
Confocal	0.0724	0.3725 [0.2122 0.4971]	Dunn's
NeuroDecon	0.0065	<0,0001	0.106 [0.09054 0.1353]
**Resolution YZ**			
STED	0.7973 [0.5553 2.203]	Dunn's	Dunn's
Confocal	<0,0001	0.5526 [0.5172 0.581]	Dunn's
NeuroDecon	>0,9999	0.0042	0.5304 [0.4382 0.6342]
**Resolution XZ**			
STED	0.6646 [0.4936 1.292]		
Confocal	0.0009	0.5423 [0.4558 0.5765]	
NeuroDecon	0.4801	0.6844	0.5919 [0.49 0.7331]
**SNR YZ**			
STED	0.574 [0.2055 0.7205]	Dunn's	Dunn's
Confocal	<0,0001	0.784 [0.766 0.798]	Dunn's
NeuroDecon	0.1046	<0,0001	0.821 [0.7833 0.83]
**SNR XY**			
STED	0.753 [0.451 0.786]	Dunn's	Dunn's
Confocal	>0,9999	0.754 [0.748 0.764]	Dunn's
NeuroDecon	0.0001	0.0002	0.798 [0.7945 0.8225]
**SNR XZ**			
STED	0.6405 [0.3463 0.7]	Dunn's	Dunn's
Confocal	<0,0001	0.762 [0.738 0.778]	Dunn's
NeuroDecon	0.4666	<0,0001	0.791 [0.7583 0.8125]

**Supplementary Fig.S7**	**Confocal**	**RLTV**	**NeuroDecon**

**Resolution XY**			
Confocal	5.292 [5.292 5.292]	Dunn's	Dunn's
RLTV	0.4072	5.292 [0.5049 5.292]	Dunn's
NeuroDecon	<0,0001	<0,0001	0.3587 [0.3468 0.3861]
**Resolution YZ**			
Confocal	3.038 [3.025 3.568]	Dunn's	Dunn's
RLTV	0.0342	4.093 [3.327 5.637]	Dunn's
NeuroDecon	<0,0001	0.0006	8.075 [5.867 12.02]
**Resolution XZ**			
Confocal	3.027 [3.008 3.459]	Dunn's	Dunn's
RLTV	0.0781	4.458 [3.217 5.493]	Dunn's
NeuroDecon	<0,0001	0.0035	10.05 [6.283 11.99]
**SNR YZ**			
Confocal	0.2181 [0.213945 0.222255]	Tukey's	Tukey's
RLTV	<0,0001	0.306 [0.299659 0.312341]	Tukey's
NeuroDecon	<0,0001	<0,0001	0.464 [0.454572 0.473428]
**SNR XY**			
Confocal	0.136 [0.135 0.162]	Dunn's	Dunn's
RLTV	0.0016	0.191 [0.1788 0.3053]	Dunn's
NeuroDecon	<0,0001	<0,0001	0.6655 [0.6255 0.7218]
**SNR XZ**			
Confocal	0.209 [0.206 0.219]	Dunn's	Dunn's
RLTV	0.0064	0.297 [0.279 0.317]	Dunn's
NeuroDecon	<0,0001	0.0048	0.48 [0.433 0.497]

**Supplementary Fig.S8c**	**Confocal**	**RLTV**	**NeuroDecon**
**Resolution**			
Confocal	0.7054 [0.5662 0.8446]	Tukey's	Tukey's
RLTV	>0,9999	0.7055 [0.5507 0.8603]	Tukey's
NeuroDecon	0.9442	0.9444	0.7637 [0.69097 0.83643]
**SNR**			
Confocal	0.5503 [0.49424 0.60636]	Tukey's	Tukey's
RLTV	0.9998	0.5485 [0.48395 0.61305]	Tukey's
NeuroDecon	0.1024	0.0994	0.7637 [0.69097 0.83643]

**Suppplementary Fig.S8d**	**Confocal**	**RLTV**	**NeuroDecon**
**SSIM for regions with average tubule width < 50 nm**			
Confocal	0.1481 [0.05691 0.23929]	Tukey's	Tukey's
RLTV	0.9228	0.09753 [0.03662 0.15844]	Tukey's
NeuroDecon	0.3156	0.1855	0.3524 [0.2336 0.4712]
**SSIM for regions with average tubule width > 50 nm**			
Confocal	0.07717 [0.0416 0.3396]	Dunn's	Dunn's
RLTV	>0,9999	0.05139]0.03134 0.2242]	Dunn's
NeuroDecon	0.5094	0.093	0.4036 [0.1408 0.5487]

Supplementary Fig.S9	RLTV	NeuroDecon
**Resolution Scaled Pearson Correlation**		
RLTV	0,8480 [0.757 0.8663]	Mann Whitney test
NeuroDecon	0.037	0,7760 [0.6528 0.816]
**Resolution Scaled Error**		
RLTV	4.468 [3.469 5.103]	Mann Whitney test
NeuroDecon	<0,0001	8.448 [7.751 10.54]

Suppplementary Fig.S10	Confocal	RLTV	NeuroDecon
**Number of segmented spines *in vitro***			
Confocal	11.4 [9.835 12.965]	Tukey's	Tukey's
RLTV	0.2642	14.7 [12.868 16.532]	Tukey's
NeuroDecon	0.2083	0.9885	15 [14.2254 15.7746]
**Number of segmented spines *in vivo***			
Confocal	16.2 [13.804 18.596]	Tukey's	Tukey's
RLTV	0.9856	15.4 [13.594 17.206]	Tukey's
NeuroDecon	0.655	0.5584	20.6 [15.36 25.84]
**Number of segmented spines in ExM**			
Confocal	9 [7.076 10.924]	Tukey's	Tukey's
RLTV	0.0981	17.4 [15.832 18.968]	Tukey's
NeuroDecon	0.0132	0.5101	21.6 [17.824 25.376]
**Spine volume *in vitro***			
Confocal	1.178 [1.0011 1.3549]	Tukey's	Tukey's
RLTV	0.003	0.5613 [0.49409 0.62851]	Tukey's
NeuroDecon	0.004	0.9927	0.5808 [0.49822 0.66338]
**Spine volume *in vivo***			
Confocal	0.4798 [0.39358 0.56602]	Tukey's	Tukey's
RLTV	0.6263	0.2157 [0.16612 0.26528]	Tukey's
NeuroDecon	0.1347	0.0467	0.994 [0.7387 1.2493]
**Spine volume *in* ExM**			
Confocal	25.67 [21.19 230.148]	Tukey's	Tukey's
RLTV	0.0996	14.9912.58317.397	Tukey's
NeuroDecon	0.0077	0.3339	7.7127.2078.217

Statistical analysis reference. The results of statistical analysis are presented as p-values at the intersections of the appropriate groups in the bottom left part of the table. The data is presented as mean [mean-SEM, mean+SEM] for data that follows a normal distribution and as median [Q1 Q3] for data that does not follow a normal distribution on the main diagonal at the intersection of the group with itself. The statistical tests used are presented at the intersections of the appropriate groups in the top right part of the table.
