# Supplementary figures and images for: NeuroDecon: A Neural Network-Based Method for Three-Dimensional Deconvolution of Fluorescent Microscopic Images
Source: Int J Mol Sci. 2025 Sep 9;26(18):8770. doi: 10.3390/ijms26188770 (PMC12469540; doi:10.3390/ijms26188770)

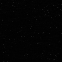

Supplement: Supplementary file 1 [file ijms-26-08770-s001.zip › ijms-3802977 NeuroDecon-data/RU-Decon dataset demo/spheres_images/100.100.500_200nm_leyka/bead_00.tif]

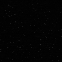

Supplement: Supplementary file 1 [file ijms-26-08770-s001.zip › ijms-3802977 NeuroDecon-data/RU-Decon dataset demo/spheres_images/100.100.500_200nm_leyka/bead_01.tif]

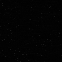

Supplement: Supplementary file 1 [file ijms-26-08770-s001.zip › ijms-3802977 NeuroDecon-data/RU-Decon dataset demo/spheres_images/100.100.500_200nm_leyka/bead_02.tif]

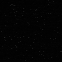

Supplement: Supplementary file 1 [file ijms-26-08770-s001.zip › ijms-3802977 NeuroDecon-data/RU-Decon dataset demo/spheres_images/100.100.500_200nm_leyka/bead_03.tif]

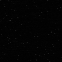

Supplement: Supplementary file 1 [file ijms-26-08770-s001.zip › ijms-3802977 NeuroDecon-data/RU-Decon dataset demo/spheres_images/100.100.500_200nm_leyka/bead_04.tif]

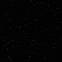

Supplement: Supplementary file 1 [file ijms-26-08770-s001.zip › ijms-3802977 NeuroDecon-data/RU-Decon dataset demo/spheres_images/100.100.500_200nm_leyka/bead_05.tif]

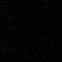

Supplement: Supplementary file 1 [file ijms-26-08770-s001.zip › ijms-3802977 NeuroDecon-data/RU-Decon dataset demo/spheres_images/100.100.500_200nm_leyka/bead_06.tif]

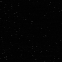

Supplement: Supplementary file 1 [file ijms-26-08770-s001.zip › ijms-3802977 NeuroDecon-data/RU-Decon dataset demo/spheres_images/100.100.500_200nm_leyka/bead_07.tif]

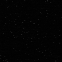

Supplement: Supplementary file 1 [file ijms-26-08770-s001.zip › ijms-3802977 NeuroDecon-data/RU-Decon dataset demo/spheres_images/100.100.500_200nm_leyka/bead_08.tif]

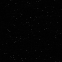

Supplement: Supplementary file 1 [file ijms-26-08770-s001.zip › ijms-3802977 NeuroDecon-data/RU-Decon dataset demo/spheres_images/100.100.500_200nm_leyka/bead_09.tif]

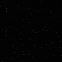

Supplement: Supplementary file 1 [file ijms-26-08770-s001.zip › ijms-3802977 NeuroDecon-data/RU-Decon dataset demo/spheres_images/100.100.500_200nm_leyka/bead_10.tif]

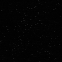

Supplement: Supplementary file 1 [file ijms-26-08770-s001.zip › ijms-3802977 NeuroDecon-data/RU-Decon dataset demo/spheres_images/100.100.500_200nm_leyka/bead_11.tif]

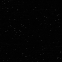

Supplement: Supplementary file 1 [file ijms-26-08770-s001.zip › ijms-3802977 NeuroDecon-data/RU-Decon dataset demo/spheres_images/100.100.500_200nm_leyka/bead_12.tif]

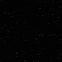

Supplement: Supplementary file 1 [file ijms-26-08770-s001.zip › ijms-3802977 NeuroDecon-data/RU-Decon dataset demo/spheres_images/100.100.500_200nm_leyka/bead_13.tif]

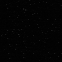

Supplement: Supplementary file 1 [file ijms-26-08770-s001.zip › ijms-3802977 NeuroDecon-data/RU-Decon dataset demo/spheres_images/100.100.500_200nm_leyka/bead_14.tif]

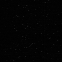

Supplement: Supplementary file 1 [file ijms-26-08770-s001.zip › ijms-3802977 NeuroDecon-data/RU-Decon dataset demo/spheres_images/100.100.500_200nm_leyka/bead_15.tif]

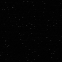

Supplement: Supplementary file 1 [file ijms-26-08770-s001.zip › ijms-3802977 NeuroDecon-data/RU-Decon dataset demo/spheres_images/100.100.500_200nm_leyka/bead_16.tif]

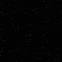

Supplement: Supplementary file 1 [file ijms-26-08770-s001.zip › ijms-3802977 NeuroDecon-data/RU-Decon dataset demo/spheres_images/100.100.500_200nm_leyka/bead_17.tif]

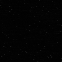

Supplement: Supplementary file 1 [file ijms-26-08770-s001.zip › ijms-3802977 NeuroDecon-data/RU-Decon dataset demo/spheres_images/100.100.500_200nm_leyka/bead_18.tif]

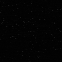

Supplement: Supplementary file 1 [file ijms-26-08770-s001.zip › ijms-3802977 NeuroDecon-data/RU-Decon dataset demo/spheres_images/100.100.500_200nm_leyka/bead_19.tif]

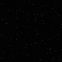

Supplement: Supplementary file 1 [file ijms-26-08770-s001.zip › ijms-3802977 NeuroDecon-data/RU-Decon dataset demo/spheres_images/100.100.500_200nm_leyka/bead_20.tif]

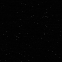

Supplement: Supplementary file 1 [file ijms-26-08770-s001.zip › ijms-3802977 NeuroDecon-data/RU-Decon dataset demo/spheres_images/100.100.500_200nm_leyka/bead_21.tif]

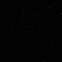

Supplement: Supplementary file 1 [file ijms-26-08770-s001.zip › ijms-3802977 NeuroDecon-data/RU-Decon dataset demo/spheres_images/100.100.500_200nm_leyka/bead_22.tif]

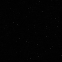

Supplement: Supplementary file 1 [file ijms-26-08770-s001.zip › ijms-3802977 NeuroDecon-data/RU-Decon dataset demo/spheres_images/100.100.500_200nm_leyka/bead_23.tif]

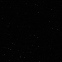

Supplement: Supplementary file 1 [file ijms-26-08770-s001.zip › ijms-3802977 NeuroDecon-data/RU-Decon dataset demo/spheres_images/100.100.500_200nm_leyka/bead_24.tif]

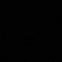

Supplement: Supplementary file 1 [file ijms-26-08770-s001.zip › ijms-3802977 NeuroDecon-data/RU-Decon dataset demo/spheres_images/16.16.200_200nm_conus_cuts/bead_00.tif]

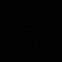

Supplement: Supplementary file 1 [file ijms-26-08770-s001.zip › ijms-3802977 NeuroDecon-data/RU-Decon dataset demo/spheres_images/16.16.200_200nm_conus_cuts/bead_01.tif]

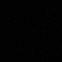

Supplement: Supplementary file 1 [file ijms-26-08770-s001.zip › ijms-3802977 NeuroDecon-data/RU-Decon dataset demo/spheres_images/16.16.200_200nm_conus_cuts/bead_02.tif]

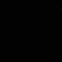

Supplement: Supplementary file 1 [file ijms-26-08770-s001.zip › ijms-3802977 NeuroDecon-data/RU-Decon dataset demo/spheres_images/16.16.200_200nm_conus_cuts/bead_03.tif]

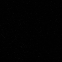

Supplement: Supplementary file 1 [file ijms-26-08770-s001.zip › ijms-3802977 NeuroDecon-data/RU-Decon dataset demo/spheres_images/16.16.200_200nm_conus_cuts/bead_04.tif]

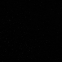

Supplement: Supplementary file 1 [file ijms-26-08770-s001.zip › ijms-3802977 NeuroDecon-data/RU-Decon dataset demo/spheres_images/16.16.200_200nm_conus_cuts/bead_05.tif]

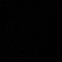

Supplement: Supplementary file 1 [file ijms-26-08770-s001.zip › ijms-3802977 NeuroDecon-data/RU-Decon dataset demo/spheres_images/16.16.200_200nm_conus_cuts/bead_06.tif]

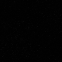

Supplement: Supplementary file 1 [file ijms-26-08770-s001.zip › ijms-3802977 NeuroDecon-data/RU-Decon dataset demo/spheres_images/16.16.200_200nm_conus_cuts/bead_07.tif]

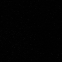

Supplement: Supplementary file 1 [file ijms-26-08770-s001.zip › ijms-3802977 NeuroDecon-data/RU-Decon dataset demo/spheres_images/16.16.200_200nm_conus_cuts/bead_08.tif]

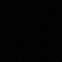

Supplement: Supplementary file 1 [file ijms-26-08770-s001.zip › ijms-3802977 NeuroDecon-data/RU-Decon dataset demo/spheres_images/16.16.200_200nm_conus_cuts/bead_09.tif]

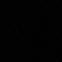

Supplement: Supplementary file 1 [file ijms-26-08770-s001.zip › ijms-3802977 NeuroDecon-data/RU-Decon dataset demo/spheres_images/16.16.200_200nm_conus_cuts/bead_10.tif]

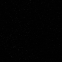

Supplement: Supplementary file 1 [file ijms-26-08770-s001.zip › ijms-3802977 NeuroDecon-data/RU-Decon dataset demo/spheres_images/16.16.200_200nm_conus_cuts/bead_11.tif]

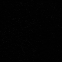

Supplement: Supplementary file 1 [file ijms-26-08770-s001.zip › ijms-3802977 NeuroDecon-data/RU-Decon dataset demo/spheres_images/16.16.200_200nm_conus_cuts/bead_12.tif]

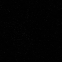

Supplement: Supplementary file 1 [file ijms-26-08770-s001.zip › ijms-3802977 NeuroDecon-data/RU-Decon dataset demo/spheres_images/16.16.200_200nm_conus_cuts/bead_13.tif]

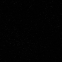

Supplement: Supplementary file 1 [file ijms-26-08770-s001.zip › ijms-3802977 NeuroDecon-data/RU-Decon dataset demo/spheres_images/16.16.200_200nm_conus_cuts/bead_14.tif]

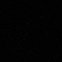

Supplement: Supplementary file 1 [file ijms-26-08770-s001.zip › ijms-3802977 NeuroDecon-data/RU-Decon dataset demo/spheres_images/16.16.200_200nm_conus_cuts/bead_15.tif]

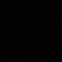

Supplement: Supplementary file 1 [file ijms-26-08770-s001.zip › ijms-3802977 NeuroDecon-data/RU-Decon dataset demo/spheres_images/16.16.200_200nm_conus_cuts/bead_16.tif]

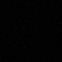

Supplement: Supplementary file 1 [file ijms-26-08770-s001.zip › ijms-3802977 NeuroDecon-data/RU-Decon dataset demo/spheres_images/16.16.200_200nm_conus_cuts/bead_17.tif]

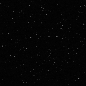

Supplement: Supplementary file 1 [file ijms-26-08770-s001.zip › ijms-3802977 NeuroDecon-data/RU-Decon dataset demo/spheres_images/19.19.200_200nm_vital/1bead_00.tif]

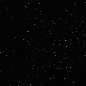

Supplement: Supplementary file 1 [file ijms-26-08770-s001.zip › ijms-3802977 NeuroDecon-data/RU-Decon dataset demo/spheres_images/19.19.200_200nm_vital/1bead_01.tif]

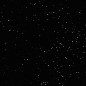

Supplement: Supplementary file 1 [file ijms-26-08770-s001.zip › ijms-3802977 NeuroDecon-data/RU-Decon dataset demo/spheres_images/19.19.200_200nm_vital/1bead_02.tif]

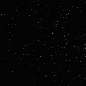

Supplement: Supplementary file 1 [file ijms-26-08770-s001.zip › ijms-3802977 NeuroDecon-data/RU-Decon dataset demo/spheres_images/19.19.200_200nm_vital/1bead_03.tif]

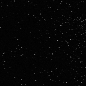

Supplement: Supplementary file 1 [file ijms-26-08770-s001.zip › ijms-3802977 NeuroDecon-data/RU-Decon dataset demo/spheres_images/19.19.200_200nm_vital/1bead_04.tif]

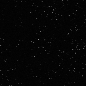

Supplement: Supplementary file 1 [file ijms-26-08770-s001.zip › ijms-3802977 NeuroDecon-data/RU-Decon dataset demo/spheres_images/19.19.200_200nm_vital/1bead_05.tif]

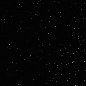

Supplement: Supplementary file 1 [file ijms-26-08770-s001.zip › ijms-3802977 NeuroDecon-data/RU-Decon dataset demo/spheres_images/19.19.200_200nm_vital/1bead_06.tif]

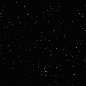

Supplement: Supplementary file 1 [file ijms-26-08770-s001.zip › ijms-3802977 NeuroDecon-data/RU-Decon dataset demo/spheres_images/19.19.200_200nm_vital/1bead_07.tif]

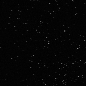

Supplement: Supplementary file 1 [file ijms-26-08770-s001.zip › ijms-3802977 NeuroDecon-data/RU-Decon dataset demo/spheres_images/19.19.200_200nm_vital/1bead_08.tif]

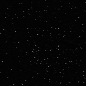

Supplement: Supplementary file 1 [file ijms-26-08770-s001.zip › ijms-3802977 NeuroDecon-data/RU-Decon dataset demo/spheres_images/19.19.200_200nm_vital/1bead_09.tif]

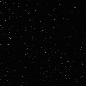

Supplement: Supplementary file 1 [file ijms-26-08770-s001.zip › ijms-3802977 NeuroDecon-data/RU-Decon dataset demo/spheres_images/19.19.200_200nm_vital/1bead_10.tif]

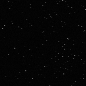

Supplement: Supplementary file 1 [file ijms-26-08770-s001.zip › ijms-3802977 NeuroDecon-data/RU-Decon dataset demo/spheres_images/19.19.200_200nm_vital/1bead_11.tif]

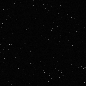

Supplement: Supplementary file 1 [file ijms-26-08770-s001.zip › ijms-3802977 NeuroDecon-data/RU-Decon dataset demo/spheres_images/19.19.200_200nm_vital/bead_00.tif]

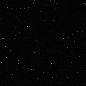

Supplement: Supplementary file 1 [file ijms-26-08770-s001.zip › ijms-3802977 NeuroDecon-data/RU-Decon dataset demo/spheres_images/19.19.200_200nm_vital/bead_01.tif]

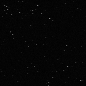

Supplement: Supplementary file 1 [file ijms-26-08770-s001.zip › ijms-3802977 NeuroDecon-data/RU-Decon dataset demo/spheres_images/19.19.200_200nm_vital/bead_02.tif]

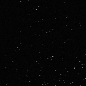

Supplement: Supplementary file 1 [file ijms-26-08770-s001.zip › ijms-3802977 NeuroDecon-data/RU-Decon dataset demo/spheres_images/19.19.200_200nm_vital/bead_03.tif]

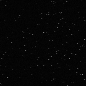

Supplement: Supplementary file 1 [file ijms-26-08770-s001.zip › ijms-3802977 NeuroDecon-data/RU-Decon dataset demo/spheres_images/19.19.200_200nm_vital/bead_04.tif]

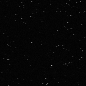

Supplement: Supplementary file 1 [file ijms-26-08770-s001.zip › ijms-3802977 NeuroDecon-data/RU-Decon dataset demo/spheres_images/19.19.200_200nm_vital/bead_05.tif]

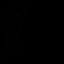

Supplement: Supplementary file 1 [file ijms-26-08770-s001.zip › ijms-3802977 NeuroDecon-data/RU-Decon dataset demo/spheres_images/22.22.100_200nm_green/bead_00_00.tif]

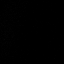

Supplement: Supplementary file 1 [file ijms-26-08770-s001.zip › ijms-3802977 NeuroDecon-data/RU-Decon dataset demo/spheres_images/22.22.100_200nm_green/bead_00_01.tif]

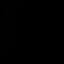

Supplement: Supplementary file 1 [file ijms-26-08770-s001.zip › ijms-3802977 NeuroDecon-data/RU-Decon dataset demo/spheres_images/22.22.100_200nm_green/bead_00_02.tif]

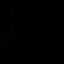

Supplement: Supplementary file 1 [file ijms-26-08770-s001.zip › ijms-3802977 NeuroDecon-data/RU-Decon dataset demo/spheres_images/22.22.100_200nm_green/bead_00_03.tif]

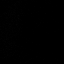

Supplement: Supplementary file 1 [file ijms-26-08770-s001.zip › ijms-3802977 NeuroDecon-data/RU-Decon dataset demo/spheres_images/22.22.100_200nm_green/bead_00_04.tif]

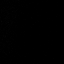

Supplement: Supplementary file 1 [file ijms-26-08770-s001.zip › ijms-3802977 NeuroDecon-data/RU-Decon dataset demo/spheres_images/22.22.100_200nm_green/bead_00_05.tif]

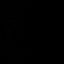

Supplement: Supplementary file 1 [file ijms-26-08770-s001.zip › ijms-3802977 NeuroDecon-data/RU-Decon dataset demo/spheres_images/22.22.100_200nm_green/bead_00_06.tif]

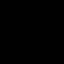

Supplement: Supplementary file 1 [file ijms-26-08770-s001.zip › ijms-3802977 NeuroDecon-data/RU-Decon dataset demo/spheres_images/22.22.100_200nm_green/bead_00_07.tif]

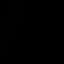

Supplement: Supplementary file 1 [file ijms-26-08770-s001.zip › ijms-3802977 NeuroDecon-data/RU-Decon dataset demo/spheres_images/22.22.100_200nm_green/bead_00_08.tif]

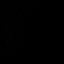

Supplement: Supplementary file 1 [file ijms-26-08770-s001.zip › ijms-3802977 NeuroDecon-data/RU-Decon dataset demo/spheres_images/22.22.100_200nm_green/bead_00_09.tif]

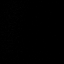

Supplement: Supplementary file 1 [file ijms-26-08770-s001.zip › ijms-3802977 NeuroDecon-data/RU-Decon dataset demo/spheres_images/22.22.100_200nm_green/bead_00_10.tif]

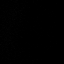

Supplement: Supplementary file 1 [file ijms-26-08770-s001.zip › ijms-3802977 NeuroDecon-data/RU-Decon dataset demo/spheres_images/22.22.100_200nm_green/bead_00_11.tif]

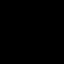

Supplement: Supplementary file 1 [file ijms-26-08770-s001.zip › ijms-3802977 NeuroDecon-data/RU-Decon dataset demo/spheres_images/22.22.100_200nm_green/bead_00_12.tif]

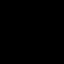

Supplement: Supplementary file 1 [file ijms-26-08770-s001.zip › ijms-3802977 NeuroDecon-data/RU-Decon dataset demo/spheres_images/22.22.100_200nm_green/bead_00_13.tif]

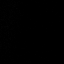

Supplement: Supplementary file 1 [file ijms-26-08770-s001.zip › ijms-3802977 NeuroDecon-data/RU-Decon dataset demo/spheres_images/22.22.100_200nm_green/bead_00_14.tif]

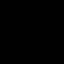

Supplement: Supplementary file 1 [file ijms-26-08770-s001.zip › ijms-3802977 NeuroDecon-data/RU-Decon dataset demo/spheres_images/22.22.100_200nm_green/bead_00_15.tif]

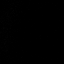

Supplement: Supplementary file 1 [file ijms-26-08770-s001.zip › ijms-3802977 NeuroDecon-data/RU-Decon dataset demo/spheres_images/22.22.100_200nm_green/bead_00_16.tif]

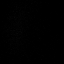

Supplement: Supplementary file 1 [file ijms-26-08770-s001.zip › ijms-3802977 NeuroDecon-data/RU-Decon dataset demo/spheres_images/22.22.100_200nm_green/bead_03_00.tif]

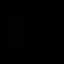

Supplement: Supplementary file 1 [file ijms-26-08770-s001.zip › ijms-3802977 NeuroDecon-data/RU-Decon dataset demo/spheres_images/22.22.100_200nm_green/bead_03_01.tif]

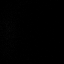

Supplement: Supplementary file 1 [file ijms-26-08770-s001.zip › ijms-3802977 NeuroDecon-data/RU-Decon dataset demo/spheres_images/22.22.100_200nm_green/bead_03_02.tif]

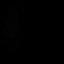

Supplement: Supplementary file 1 [file ijms-26-08770-s001.zip › ijms-3802977 NeuroDecon-data/RU-Decon dataset demo/spheres_images/22.22.100_200nm_green/bead_03_03.tif]

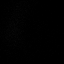

Supplement: Supplementary file 1 [file ijms-26-08770-s001.zip › ijms-3802977 NeuroDecon-data/RU-Decon dataset demo/spheres_images/22.22.100_200nm_green/bead_03_05.tif]

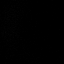

Supplement: Supplementary file 1 [file ijms-26-08770-s001.zip › ijms-3802977 NeuroDecon-data/RU-Decon dataset demo/spheres_images/22.22.100_200nm_green/bead_03_06.tif]

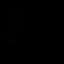

Supplement: Supplementary file 1 [file ijms-26-08770-s001.zip › ijms-3802977 NeuroDecon-data/RU-Decon dataset demo/spheres_images/22.22.100_200nm_green/bead_03_07.tif]

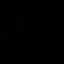

Supplement: Supplementary file 1 [file ijms-26-08770-s001.zip › ijms-3802977 NeuroDecon-data/RU-Decon dataset demo/spheres_images/22.22.100_200nm_green/bead_03_08.tif]

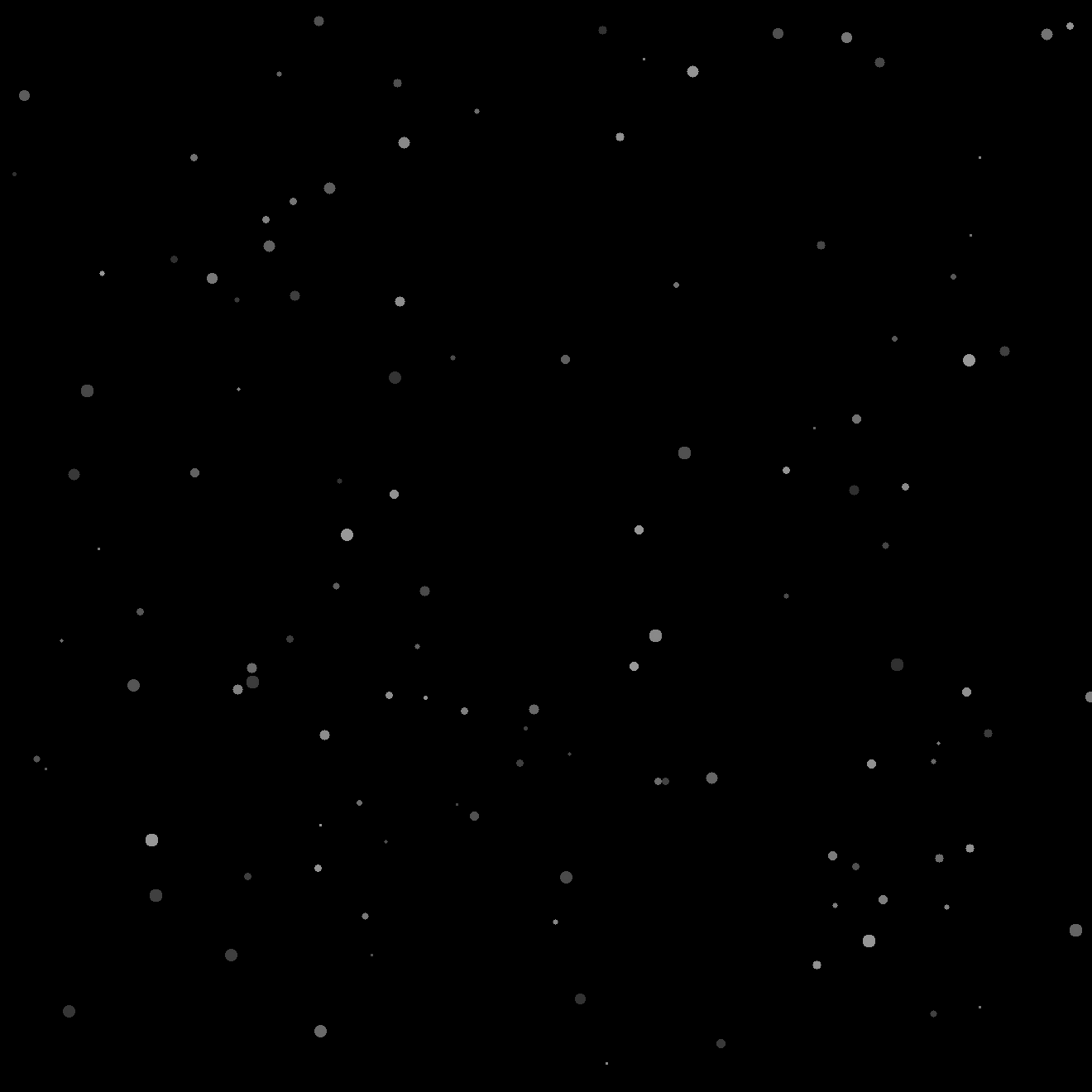

Supplement: Supplementary file 1 [file ijms-26-08770-s001.zip › ijms-3802977 NeuroDecon-data/RU-Decon dataset demo/synthetic_data/synthetic_spheres.tiff]

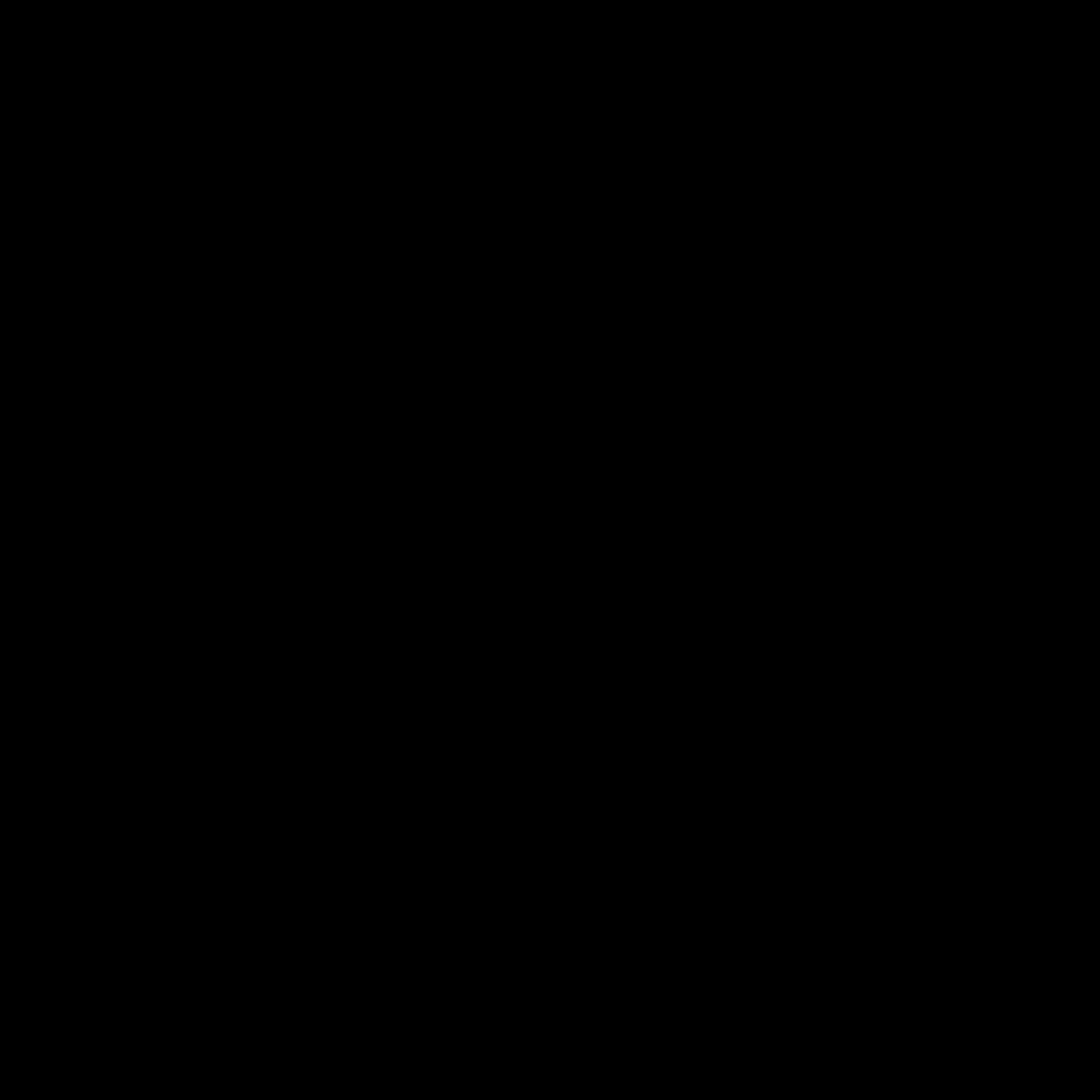

Supplement: Supplementary file 1 [file ijms-26-08770-s001.zip › ijms-3802977 NeuroDecon-data/RU-Decon dataset demo/synthetic_data/synthetic_sticks.tiff]
